# Different Cell Wall Compositions of ESKAPE Isolates on Glass Surfaces Impact Adhesion Adaptability to Dynamic Shear Stress

**DOI:** 10.3390/microorganisms14030623

**Published:** 2026-03-10

**Authors:** Zhuoyi Cui, Anje M. Slomp, Alesia V. Quiroga, Jelly Atema-Smit, Hans J. Kaper, Brandon W. Peterson

**Affiliations:** Department of Biomaterials and Biomedical Technology, University Medical Centre Groningen, University of Groningen, 9713 AV Groningen, The Netherlands

**Keywords:** ESKAPE, bacterial cell wall, shear rate, adhesion, spring constant, adaptation, recovery/retention, directionality index

## Abstract

Although many studies have focused on the initial adhesion of bacteria, there have been few that looked at responses to changing environmental conditions. To more closely examine the viscoelastic nature of initial adhesion, surface-associated bacteria were quantified and monitored for their Brownian motion vibrations. This study used a flow chamber to observe the surface association of *Enterobacter cloacae BS* 1037, *Staphylococcus aureus ATCC* 12600, *Klebsiella pneumoniae*–1, *Acinetobacter baumannii*–1, *Pseudomonas aeruginosa PA O*1, and *Enterococcus faecalis* 1396 to glass under dynamic shear rates of 7–15–30 s^−1^, 15–30–60 s^−1^, and 30–15–7 s^−1^. Comparing increasing and decreasing shear rates, information about retention and recovery became apparent. Coccoid bacteria primarily reacted to directional changes in shear rates with changes in either surface-associated bacterial densities or surface-associated strength independently. *A. baumannii* and *E. faecalis* did not change their associated strength, whereas *S. aureus* did not change its associated density. Bacillus bacteria demonstrated differences in both associations with directional changes in shear rates. We demonstrate that retention and recovery are different methods of adaptation to environmental conditions utilised by different bacterial species. These adaptations may form the basis of upregulation and downregulation responses used for survival.

## 1. Introduction

Bacterial infections have become a significant public health challenge [1]. Numerous infections associated with ESKAPE pathogens—namely *Enterococcus faecium*, *Staphylococcus aureus*, *Klebsiella pneumoniae*, *Acinetobacter baumannii*, *Pseudomonas aeruginosa*, and *Enterobacter* spp.—are closely related to their capacity to adhere to medical device surfaces [2,3,4]. For example, *Staphylococcus aureus* exhibits a strong ability to adhere to orthopaedic implant materials such as titanium, cobalt–chromium alloys, and polyethene, leading to the formation of biofilms that are difficult to eliminate [5,6]. Similarly, *Pseudomonas aeruginosa* tends to colonise the surfaces of urinary catheters, with latex catheters facilitating quicker adhesion and biofilm development compared to PVC or silicone catheters [7,8,9].

Bacterial adhesion related to medical devices poses significant challenges in hospitals and water systems. It can lead to the failure of medical implants and the rise in costs associated with healthcare. Developing more effective strategies to predict and prevent bacterial adhesion can enhance medical device performance and reduce the risk of infection [10].

Bacteria detect potentially disadvantageous factors resulting in stress responses to survive [11]. The ESKAPE panel of bacteria is well documented for its ability to develop antimicrobial resistance, and thus was selected for its adaptability. A crucial key for bacterial sustainability in the environment is their ability to acclimate to the stressful conditions, including alterations in pH [12], temperature [13], nutrient utility [14], oxygen content [15], and osmotic pressure gradients [16,17]. Microbes detect their surroundings via various sensors and receptors, which translate into appropriate cellular response(s) that are optimal for survival [18]. While diverse environmental stimuli have been examined for their influence on microbes, the effects of bacterial changes in mechanical and/or physical forces have become popular, as they are determinants of bacterial survival and important factors for exploring antimicrobial methods [19,20,21,22,23].

In recent years, there has been an expeditious expansion of the understanding of bacterial behaviours in fluid flow [24]. For example, the relationship between how bacteria sense flow and surface contact has been widely studied [25,26,27,28,29]. Also, there is great value in comparing how bacteria sense and respond to flow in general [30,31], as flow plays a major role in the formation of biofilms [28]. Certain mechanical stimuli affect the physiology of microorganisms [21,23,32]. Recent studies have also demonstrated that microorganisms can sense and respond to modifications in culture conditions when grown in the buoyant, low-fluid-shear environment of microgravity [20,23,33,34]. Moreover, it has been hypothesised that cells sense changes in shear at the surface [35]. However, these studies mainly investigate independent shear rate conditions, lacking analysis of potential adaptation to changing environments.

While changes in the physical forces of fluid shear play a crucial role in evolution and microbial physiology [36], it remains unclear how microbial cells convert these mechanical signals into their behaviour and whether they adapt to these changes. Based on the current literature, several bacterial species, including *Escherichia coli* (via FimH catch-bond mechanism), *Staphylococcus aureus*, and *Pseudomonas aeruginosa*, have been shown to resist hydrodynamic shear forces through distinct mechano-sensing and adhesion strategies [37,38,39,40]. However, most existing studies have employed steady-state shear conditions exceeding 100 s^−1^ to model arterial or industrial regimes [41], leaving a critical gap in understanding bacterial adhesion under dynamic shear trajectories within the microcirculation-relevant range (7–60 s^−1^) where physiological flow fluctuations are inherent [39,42]. Therefore, this study employs both increasing (7→15→30 s^−1^; 15→30→60 s^−1^) and decreasing (30→15→7 s^−1^) shear rate changes to systematically investigate how directional changes to shear rates modulate bacterial association to the surface. The design addresses adaptation in adhesion properties with directional changing shear rates under conditions where physiologically relevant flow fluctuations occur.

The aim of this study is to determine whether different bacterial species can sense mechanosensitive changes in their environment to trigger a survival reaction. The removal of environmental sensing may lead to the inability to react and potentially develop resistance. Thus, the experimental approach focuses on early (pre-mutation and pre-regulation changes) association with a surface under short time periods. Bacteria associated with the surface are quantified and imaged at 25 frames per second for 20 s to observe Brownian motion or confined Brownian motion, if attached, considered as vibrations over the surface. Bacteria associated with the surface interact via a combination of chemical interactions of extracellular molecules, cell wall appendages, and strengthen the adhesion bond with cell wall deformation [25]. These collective interactions have been previously classified as “tethers” in which the total adhesion bond strength is reflected as the sum total of all the attached tethers [43]. The extracellular molecules are polymeric, and the cell wall encapsulates a fluid environment, providing a viscoelastic interaction with the surface. By tracking the location of bacteria within the series of images, the observed vibrations were analysed, and the associated spring constant was calculated. The associated spring constant was determined for the elastic portion of the viscoelastic nature of the association to the surface.

To determine the nature of any adaptation, a single imaging window was monitored while shear rates were sequentially increased, providing the bacteria with additional energy. For measurements, the shear was arrested, eliminating additional energy during imaging before increasing the shear rate to the next rate in sequence. Surface-associated bacteria here are said to be retained with exposure to higher shear rates. Conversely, shear rates were also decreased sequentially to examine recovery from higher energy shear rates. The ratio between recovery and retention was determined to highlight directional adaptation patterns between the six tested bacterial strains.

## 2. Materials and Methods

### 2.1. Bacterial Strains, Growth Conditions, and Harvesting

Six bacterial strains were selected for this study and grown from stock solutions (7% DMSO, kept at −80 °C) on blood agar plates at 37 °C for 24 h. Details on the type and shape of the bacterial strains are listed in Table 1.

A single bacterial colony was transferred into 10 mL of TSB (Tryptone Soya Broth, OXOID, Basingstoke, UK, 500 g) preculture and incubated for 24 h at 37 °C before transfer into 200 mL TSB main cultures, incubated at 37 °C. After 16 h, bacteria were harvested by centrifugation three times at 5000× *g* for 5 min. Between centrifugation cycles, the bacteria were washed and re-suspended in PBS buffer (phosphate-buffered saline, 0.5 M (K_2_HPO_4_, KH_2_PO_4_), 0.15 M NaCl (8.76 g/L), pH 7.0). After the final wash, the bacteria were suspended in PBS buffer and sonicated in an ice/water bath for 3 × 10 s (Vibra Cell model 375; Sonics and Materials, Danburry, CT, USA) to break up aggregates, with 30 s breaks in between cycles. The final experimental bacterial suspension was then adjusted to 3 × 10^8^ mL^−1^ in PBS using a Bürker-Türk counting chamber.

### 2.2. Flow Chamber

**Figure 1:**
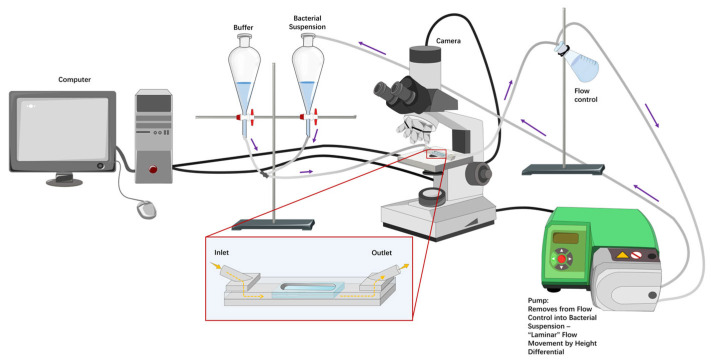
The schematic devices of bacteria in the flow system. Diagram of the system comprising a flow chamber set on the stage of an upright microscope connected to a Balser CCD camera and a desk computer, a flow pump, and connectors, plus various tubes. The containers provide PBS buffer or bacterial suspensions that flow through the inlet of the homemade flow chamber. The outlet of the flow chamber empties into a flow control container that has equal vertical height as the buffer/bacterial suspension containers to control the flow rate (not depicted as equal for clarity of components). The pump removes the calibrated volume from the flow control container, putting it back into the corresponding container (buffer for setup and bacterial suspension during experiments). The height differential then promotes “laminar” flow movement through the chamber at the rate removed by the pump, forming a circulating system. Purple arrows indicate the movement of fluid through the tubing. The insert image shows an enlarged depiction of a microfluidic device on the microscope stage. Yellow arrows indicate the movement of fluid into and out of the microfluidic device. The lighter grey shape depicts the flow chamber, while the lighter blue shape depicts two microscope slides. A flow pump can adjust the flow rate in a microfluidic system. The flow pump was used to replace the bacterial solution at shear rates of 7 s^−1^, 15 s^−1^, 30 s^−1^, and 60 s^−1^, corresponding to flow rates of 0.63 mL/min, 1.35 mL/min, 2.72 mL/min, and 5.45 mL/min, respectively. The camera and computer were used to capture images through the glass slides for adhesion on the bottom surface.

**Figure 2:**
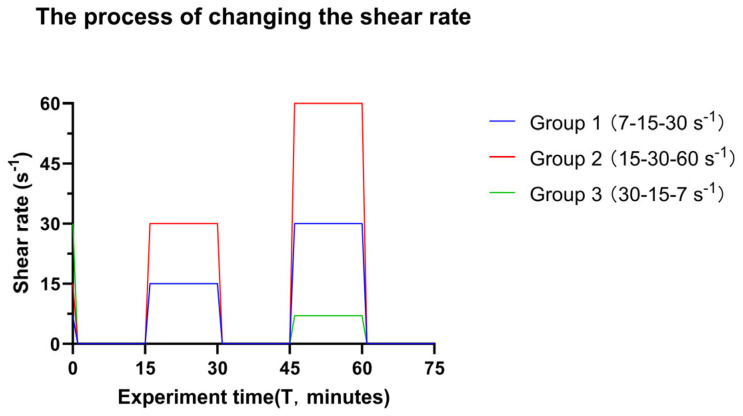
The timescale of changing the shear rate in the experiment. The bacterial suspension was transported over the glass surface at the initial shear rate until adequate surface coverage of surface-associated bacteria was detected, reflecting approximately 1 × 10^6^ bacteria cm^−2^. At this point, the shear was arrested, and images were taken (Time = 0 min). Sequential images were taken from the middle of the flow chamber to create videos. After video capture, the intermediate shear rate was introduced for 15 min and subsequently arrested. New videos were captured, and the final shear rate was introduced. The different colour lines indicate different groups of transitional shear rates. Of note, Groups 1 and 3 have an overlap at the immediate shear rate and time.

The homemade parallel plate flow chamber (Figure 1) was 17.5 cm long, 1.6 cm wide, and had a height of 0.075 cm. The flow chamber has been previously described in detail [45]; briefly, stainless steel top and bottom plates have a depression that fits a glass microscope slide, creating a flat surface for fluid to move over. The plates are screwed together with 12 screws with a Teflon insert (height 0.075 cm) and have inlet and outlet spouts to attach tubing, attached to the plates with 4 additional screws. The glass slides and spouts are sealed with rubber O-rings to prevent leakage. A series of flow rates was set for each treatment group according to the initial, intermediate, and final shear rates in Table 2 (flow pump, WATSON-MARLOW, type 520S). Bacteria were monitored for adhesion in a 1392 × 1040 window at the middle of the flow chamber using an upright microscope (Olympus microscope, type BH-2). Adhesion continued under the initial shear rate until an approximate bacterial density of 1 × 10^6^ cells/cm^2^ was observed, at which time the flow was arrested for 15 min. Then, sequential images (25 frames/s for 20 s) of surface-associated bacteria were recorded with a Balser CCD camera (type A102, Figure 1), for image capture, and a PC (Figure 1) with a FireWire interface to control the camera. After collecting the images, the intermediate shear rate level was applied for 15 min, followed by a 15 min arrest period before capturing new sequential images. This process was then repeated for the final shear rate. The timescale for changing the shear rate is summarised in Figure 2. The sequential images were analysed by an image analysis programme (Matlab, The MathWorks, version 2023a, Natick, MA, USA). Each image (1392 × 1040) with an 8-bit grey scale was diminished to 1392 × 200 pixels. Surface-associated density was expressed as cells per cm^2^ when sequential images displayed no increase in surface-associated density (reaching a plateau).

All experiments were performed at a single calibrated temperature to ensure cross-strain comparability; reported effects reflect relative differences under identical thermal conditions [46]. Three biological replicate experiments were performed for each bacterial strain at three different shear rates and the same room temperature (22 °C).

**Table 2:** Experimental shear rate setting and analysis data grouping.

	Group Number	Initial Shear Rate (s^−1^)	Intermediate Shear Rate (s^−1^)	Final Shear Rate (s^−1^)	The Trend of Shear Rate	Comparison Basis
Treatment Group	1	7	15	30	↑	various shear rates, time
	2	15	30	60	↑	various shear rates, time
	3	30	15	7	↓	various shear rates, time
		Comparison Group			Comparison basis	
Analysis Group	4	Groups 1 and 3			7 s^−1^, time	
	5	Groups 1 and 2, Groups 2 and 3			15 s^−1^, time	
	6	Groups 1, 2 and 3			30 s^−1^, time	
	7	Groups 1 and 3			15 s^−1^, same time	

Treatments with increasing shear rate are designated with ↑, while treatments with decreasing shear rate are designated with ↓. Group 1 simulates gradual flow intensification and assesses bacterial adhesion stability under accumulating hydrodynamic shear. Group 2 probes a higher limit of shear rate (still clinically relevant conditions where physiologically relevant flow fluctuations occur) to determine if a viscoelastic failure may cause a bacterial response. Group 3 investigates potential relaxation under reduced shear rates. Shear rates were selected to cover a range from low to high hydrodynamic stress relevant to microscale physiological environments [47] (e.g., venous to near-arterial regimes), as previous studies have demonstrated that hydrodynamic forces significantly influence bacterial adhesion during the initial stages of surface colonisation [48]. Groups 4, 5, 6, and 7 categorised the experimental data, isolating variables for control analysis. Groups 4, 5, and 6 are analysed at the same shear rate (7, 15, and 30 s^−1^) with different times; Group 7 is analysed at the same shear rate (15 s^−1^) and the same time. The flow rate corresponding to shear rates of 7 s^−1^, 15 s^−1^, 30 s^−1^, and 60 s^−1^ is, respectively, 0.63 mL/min, 1.35 mL/min, 2.72 mL/min, and 5.45 mL/min. Time reflects the elapsed time to shear rates for bacteria that may or may not be surface-associated. Detachment and reattachment events are quantified for surface-associated counts, but are not included in spring constant analysis, unless the detachment/reattachment remained in the confined analysis region.

### 2.3. Microscope Glass Cleaning

All experiments were carried out on microscope glass slides (Menzel GmbH, Braunschweig, Germany) with dimensions 76 × 26 × 1 mm. Glass surfaces were cleaned to remove debris and oils. The glass slides were submerged in 2% RBS solution (Omniclean RBS 35, Breda, The Netherlands) and sonicated for 5 min. Then, the slides were rinsed with running hot tap water, dipped in methanol, and rinsed thoroughly with demineralised water. The slides were not dried as they would be hydrated with PBS upon flow chamber assemblage. Both the upper and lower glass slides were replaced for each biological replicate.

### 2.4. Data Analysis

Microscopic images were acquired at fixed positions (did not change between shear rate changes) within the flow cell using identical imaging parameters across all experiments. Each image had a size of 1392 × 200 pixels, corresponding to a constant field-of-view area for all conditions. For each condition, surface-associated densities were quantified from 3 biological replicates. Surface-associated bacteria were quantified using image-based cell counting. Identical thresholds and analysis algorithms were applied across all images; attachment density was expressed as cells per cm^2^. For each shear-rate step, 500 images were captured at 25 frames per second. Surface-associated density was determined at the first instance when the counts reached a stable plateau, defined as a period after constant shear conditions were applied (and a 15 min arrest period), during which no systematic change in surface-associated density was observed over time. Plateau phases at identical shear rates were determined independently for each shear rate condition and biological replicate.

During acquisition, this study tracked and analysed the video sequences frame by frame and extracted the minute vibrations generated by Brownian motion or confined Brownian motion. These vibrations, partly impacted by shear rate history, are presented as a sequence of displacement changes over time, directly reflecting the response behaviour of bacteria to the dynamic external environment. This displacement was fitted to the mean squared displacement (MSD) as a function of time (t) using a classical elastic model [49]. The vibrational behaviour of bacteria can be considered as the sum of multiple interactions (tethers) between the bacteria and the glass surface. Under a short time period, these are mathematically dominated by some equivalent elastic constraint (such as the elasticity of the cell wall, the rigidity of the cytoskeleton, etc.). As such, Ks should be interpreted as a parameter derived from an approximation of the bacterial mechanical response under a specific experimental regime and fitting window. Its physical meaning is therefore closer to an effective measure of confinement strength of bacterial fluctuations, rather than an intrinsic material constant of a bacterium. Consequently, the numerical value of Ks is only meaningful within the timescale over which the MSD fitting is performed. The analysis excluded non-individual bacteria that adhered and clustered together during the observation period, bacteria that jumped and spun in the non-adherent state, and bacteria that moved away from the surface during and at the end of the observation period. The specific number of bacteria selected based on the video and the conditions described above is shown in Table 3. The selected bacteria account for 84% [range 73–92%] of the total adhesion number of bacteria (1392 × 200 pixels). In summary, this study carefully characterised the surface-associated density and movement of 6 different bacterial strains under different shear rate changes. The stronger the association to the surface, the more confined the Brownian motion will be. The Brownian motion is modelled via mean squared displacement (MSD, (Equation (1))), which is then used to calculate the spring constant (Ks). The stronger the association bond between the bacteria and the surface becomes, the stiffer the resulting spring constant will be.

Videos were analysed using MATLAB software to capture the trajectory coordinates of the centre of mass for each bacterium over time. These coordinates were used to calculate the bacterial average mean square displacement (MSD) over time and fit to a regression curve Formula (1) via GraphPad Prism (Version 10). ImageJ (Fiji-win64) software was used to quantify the number of surface-associated bacteria. After data processing, comparisons of total bacterial counts and the bacterial spring constant were performed using one-way analyses of variance (ANOVA) with Tukey’s HSD post hoc test via GraphPad Prism with alpha set to 0.05, resulting in *p* < 0.05 as significance for all tests. As a result of the high N for spring constants, scientific significance was deemed at *p* < 0.001.

The MSD as a function of time is indicative of the average position of a single bacterium and was calculated [49] at different time points according to

MSD = ⟨⟨Δr^2^⟩⟩= (1/10)Σ (k = 1 to 10)(1/N)Σ (i = 1 to N) (rᵢ − rⱼ)^2^ (1)

To measure how much the bacteria move over time, the average distance the bacteria travelled from their starting points was calculated over 10 s. This study used ten different starting positions (i.e., starting t = 0 s to ending t = 10 s and finishing with starting at t = 10 s and ending at t = 20 s) to make sure the results are reliable and not influenced by any one local area. For each starting location rᵢ (position of the particle at the i time point), the analysis tracked how far the bacteria moved at 250 time points (at 25 frames per second). We then compared the position at each time point to its initial position rⱼ (j = i, i + 1, …, N) and calculated how much it had shifted, (rᵢ − rⱼ)^2^ at each time step relative to the reference position. ⟨⟨Δ*r*^2^⟩⟩ indicates averaging across both multiple starting locations and multiple time points. By averaging these shifts across both time and starting locations, we obtained a single overall measure that reflects how the bacterium moved in the system.

To obtain the spring constant (Ks), the MSD Equation (2) below was used.

MSD = A(1(−exp(−t/B)) + C(1 − exp)[(−t/(2B))]^2^) (2)

Nonlinear regression in GraphPad Prism was used to generate a regression curve and extract parameters. Parameters A, B, and C were simplified to allow GraphPad to generate the regression curves. Each parameter simplification had practical meaning: A = kT/Ks (provides information about the balance of thermal energy and substrate interaction strength), B = τ (characteristic timescale of the system, reflecting the transition from rapid to slower motion), and $C=\delta_{1}^{2}$ (squared displacement at t = 0, describing the initial displacement variability). Then, the spring constant was calculated from A = kT/Ks, with K being the Boltzmann constant (1.38 × 10^−23^ JK^−1^) and T set to 298.15 K.

For each shear-rate step, surface-associated density was determined, where no systematic changes in counts were observed with time. These phases were determined independently for each shear rate condition per biological replicate. To assess changes in behaviour from the initial shear rate to the final shear rate, the ratio of surface-associated density was calculated. For increasing shear rates, this is classified as the retention index, as bacteria remain associated with the surface under higher shear rates and associated energy. For decreasing shear rates, this is classified as the recovery index, as bacteria can relax and recover under lower shear rates and associated energy.

The low-shear reference density (N_7_) was the surface-associated bacterial density at 7 s^−1^ after 15 min before shear modulation. The Retention Index (RI) quantitatively evaluates the ability of bacteria to remain associated with a surface after experiencing dynamic shear rate increases. It assesses bacterial retention capacity under progressively increasing hydrodynamic-associated energy.

For Group 1 (7–15–30 s^−1^), the retention index was defined as:

(3) $${RI}_{30}=\frac{N_{30}}{N_{7}}$$

For Group 2 (15–30–60 s^−1^), the retention index was defined as:

(4) $${RI}_{60}=\frac{N_{60}}{N_{15}}$$

The Recovery Index (RI_Down_) quantitatively evaluates the extent to which bacterial association to a surface recovers after exposure to a high shear rate followed by a return to a lower shear rate condition.

For Group 3 (30–15–7 s^−1^), the recovery index was defined as:

(5) $${RI}_{Down}=\frac{N_{7}}{N_{30}}$$

Bacteria may have a stronger affinity to adapt via retention or recovery. The Directionality Index (DI) is the ratio between the recovery index and retention index. A value greater than 1 favours recovery, while a value less than 1 favours retention. This value is only meaningful under the condition that the starting and ending shear rates are inversely the same (Group 3 recovery and Group 1 retention).

The directionality index (DI) was calculated as:

(6) $$DI=\frac{{RI}_{Down}}{{RI}_{30}}$$

Retention, recovery, and directionality indices were calculated for each biological replicate individually. Median values were subsequently computed for visualisation. Surface-associated densities and spring constants were log_10_-transformed to account for heteroscedasticity, whereas retention and recovery indices were presented on their original ratio scales. Directional indices were log_10_-transformed to set the inflection point at zero.

Retention, recovery, and directionality indices are displayed as dot plots showing individual biological replicate values as single point estimates and the median values. Reference lines for the inflection point were set at RI = 1 for retention and recovery indices. The directional indices were log_10_-transformed to make the reference line at DI = 0. This allows for a quadrant analysis where each quadrant has distinct affinities for retention or recovery. All figures were generated using GraphPad Prism. For all indices, each biological replicate was represented by a single surface-associated count per shear condition and mean spring constant as a single point estimate.

## 3. Results

The ESKAPE panel of bacteria has different cell shapes and cell wall compositions as seen by Gram stain (Table 1). The different combinations of shape and cell wall composition provide ample differences from which similar adaptation patterns could be used to reflect on more general adaptation mechanisms than species-specific mechanisms.

### 3.1. Surface-Associated Bacterial Density for ESKAPE Panel Species on Glass Surfaces Adapting to Various Shear Rates

**Figure 3:**
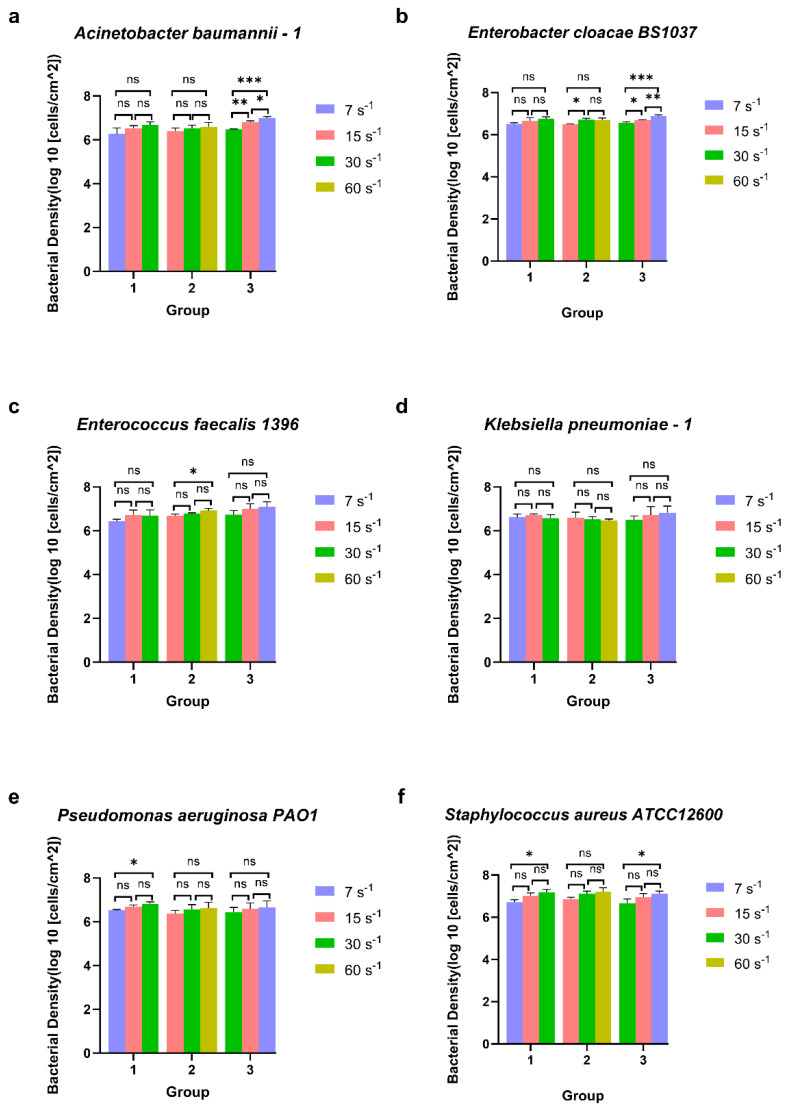
The surface-associated density of ESKAPE panel bacteria under three groups of different shear rates. Six bacterial species *Acinetobacter baumannii*–1 (**a**), *Enterobacter cloacae* BS1037 (**b**), *Enterococcus faecalis* 1396 (**c**), *Klebsiella pneumoniae*–1 (**d**), *Pseudomonas aeruginosa* PA01 (**e**), and *Staphylococcus aureus* ATCC 12600 (**f**) on glass were exposed to three treatment groups according to the directional change in shear rate, namely group 1 (7 s^−1^→15 s^−1^→30 s^−1^), group 2 (15 s^−1^→30 s^−1^→60 s^−1^), and group 3 (30 s^−1^→15 s^−1^→7 s^−1^). Mean surface-associated densities are represented with error bars as standard deviation (SD, n = 3 biological replicates). Data were analysed using a one-way ANOVA with Tukey’s HSD post hoc test. Statistical significance is represented by * *p* < 0.05, ** *p* < 0.01, *** *p* < 0.001. Representative images of significant differences can be found in Figure S1.

**Figure 4:**
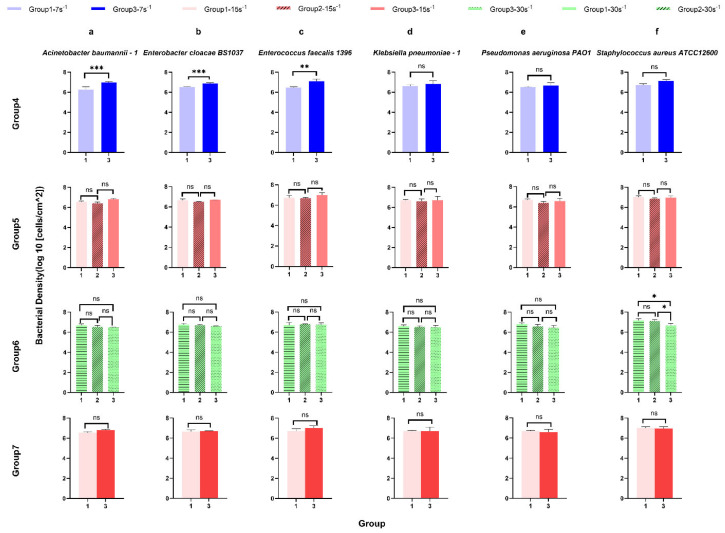
The surface-associated density of ESKAPE panel bacteria on the glass surface under the same shear rate for control analysis. Six bacterial species *Acinetobacter baumannii*–1 (**a**), *Enterobacter cloacae* BS1037 (**b**), *Enterococcus faecalis* 1396 (**c**), *Klebsiella pneumoniae*–1 (**d**), *Pseudomonas aeruginosa* PA01 (**e**), and *Staphylococcus aureus* ATCC 12600 (**f**) on glass were exposed to three treatment groups according to the directional change in shear rate, namely group 1 (7 s^−1^→15 s^−1^→30 s^−1^), group 2 (15 s^−1^→30 s^−1^→60 s^−1^), and group 3 (30 s^−1^→15 s^−1^→7 s^−1^). The data were further divided into three groups (rows, groups 4–7) according to the same shear rate, namely group 4 (7 s^−1^), group 5 (15 s^−1^), and group 6 (30 s^−1^). Group 7 showed the bacterial density of six bacterial strains at the same shear rate (15 s^−1^) and the same time (intermediate). Mean surface-associated densities are represented with error bars as standard deviation (SD, n = 3 biological replicates). Data were analysed using a one-way ANOVA with Tukey’s HSD post hoc test. Statistical significance is represented by * *p* < 0.05, ** *p* < 0.01, *** *p* < 0.001.

Bacteria were subjected to directional changes in shear rate in a parallel plate flow chamber over time during their association with glass and monitored for their surface association bacterial density. To decipher adaptation effects from changing shear rates, three treatment groups were established. Within these three treatment groups, each treatment group underwent three biological replicate experiments, and four additional analysis groups were tested as controls (Table 2). An ANOVA test with Tukey’s HSD post hoc test was employed to compare the differences in surface-associated bacterial densities of six bacterial species under three different shear rate conditions (Figure 3 and Figure 4). There was a significant increase in surface-associated bacterial densities between *Pseudomonas aeruginosa PA O*1 (Figure 3e) and *Staphylococcus aureus ATCC* 12600 (Figure 3f) in group 1 (7 s^−1^→15 s^−1^→30 s^−1^). The other four bacterial species had no significant change in surface-associated bacterial density under any conditions in group 1.

In group 2 (15 s^−1^→30 s^−1^→60 s^−1^), there was a significant increase in the surface-associated bacterial density of *Enterococcus faecalis* 1396 (Figure 3c) from 15 s^−1^ to 60 s^−1^. *Enterobacter cloacae BS* 1037 (Figure 3b) significantly enhanced surface-associated bacterial density from 15 s^−1^ to 30 s^−1^, but the significant difference was not maintained when the shear rate increased to 60 s^−1^. There was no difference in the surface-associated bacterial density between *Acinetobacter baumannii*–1 (Figure 3a), *Klebsiella pneumoniae*–1 (Figure 3d), *Pseudomonas aeruginosa PA O*1 (Figure 3e), and *Staphylococcus aureus ATCC* 12600 (Figure 3f) in group 2.

With decreasing shear rates, *Acinetobacter baumannii*–1 (Figure 3a) *and Enterobacter cloacae BS* 1037 (Figure 3b) demonstrated a significant increase in surface-associated bacterial density between all shear rates in group 3 (30 s^−1^→15 s^−1^→7 s^−1^). Also, the density of *Staphylococcus aureus ATCC* 12600 (Figure 3f) displayed a significant increase in group 3 with no significance independently from 30 s^−1^ to 15 s^−1^ and from 15 s^−1^ to 7 s^−1^. The other three bacterial strains did not significantly change in surface-associated bacterial density in group 3.

### 3.2. Surface-Associated Bacterial Density for ESKAPE Panel Species on Glass Surfaces Controlled at the Same Shear Rates

Using the same ANOVA and post hoc tests in groups 1–3, thus controlling for multiple comparisons, four additional control assessments were compared to determine the influences of potential confounding variables. Group 4 contains all test conditions with a shear rate of 7 s^−1^, group 5 has a constant shear rate of 15 s^−1^, and group 6 has a constant shear rate of 30 s^−1^. Group 7 controls both shear rate and elapsed time with 15 s^−1^ as the intermediate assessment, comparing increasing and decreasing shear adaptation (Figure 4). In group 4 (7 s^−1^ shear rate), the surface-associated bacterial density of *Acinetobacter baumannii*–1 (Figure 4 group 4), *Enterobacter cloacae BS* 1037 (Figure 4 group 4), and *Enterococcus faecalis* 1396 (Figure 4 group 4) increased significantly between treatment group 1 (un-adapted: 7 s^−1^→15 s^−1^→30 s^−1^) and group 3 (reduced shear: 30 s^−1^→15 s^−1^→7 s^−1^), while the surface-associated bacterial density for the other three bacterial species did not change significantly. Interestingly, there was no significant difference in the surface-associated bacterial density of ESKAPE bacteria (Figure 4 group 5) with a 15 s^−1^ shear rate, demonstrating that the adhesion density did not significantly change in different shifts in shear rate and elapsed time or when elapsed time was held constant (group 7). Furthermore, only the surface-associated bacterial density of *Staphylococcus aureus ATCC* 12600 (Figure 4 group 6 f) raised significantly under 30 s^−1^ when more time to adhere was allowed and adaptation from lower shear rates was permitted [group 1, final: (7 s^−1^→15 s^−1^→30 s^−1^) and group 3, un-adapted (30 s^−1^→15 s^−1^→7 s^−1^), group 2, intermediate (15 s^−1^→30 s^−1^→60 s^−1^) and group 3, un-adapted (30 s^−1^→15 s^−1^→7 s^−1^)]. The other five bacterial species did not show significant differences in surface-associated bacterial density with time at a 30 s^−1^ shear rate.

Across the three defined shear rate groups (7–15–30 s^−1^, 15–30–60 s^−1^, and 30–15–7 s^−1^), surface-associated bacterial density measurements revealed pronounced strain-specific differences both within individual groups and between groups. However, comparisons of bacterial density alone do not capture how surface-associated bacterial density responds to the direction of shear change along each group. We therefore next quantified retention, recovery, and direction-dependent attachment behaviour under these continuous shear rates (Figure 4).

### 3.3. Strain-Specific Surface-Associated Bacterial Densities Under Directional Dynamic Shear

**Figure 5:**
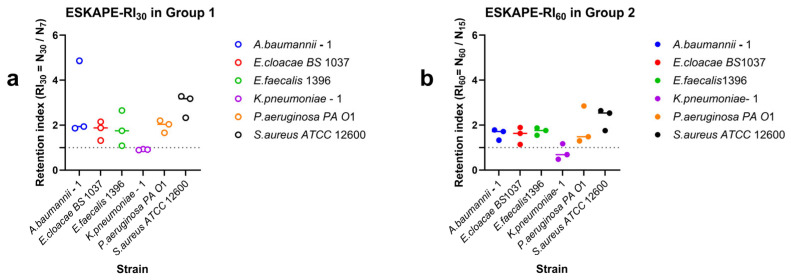
Retention of surface-associated densities along increasing shear rates. Retention indices were calculated from surface-associated densities measured along the 7–15–30 s^−1^ (**a**) and 15–30–60 s^−1^ (**b**) shear rates. Individual points represent biological replicates, while the horizontal line represents the median value. The dashed horizontal line indicates RI = 1, corresponding to no change in surface-associated bacterial densities between the final shear rate and the initial shear rate.

**Figure 6:**
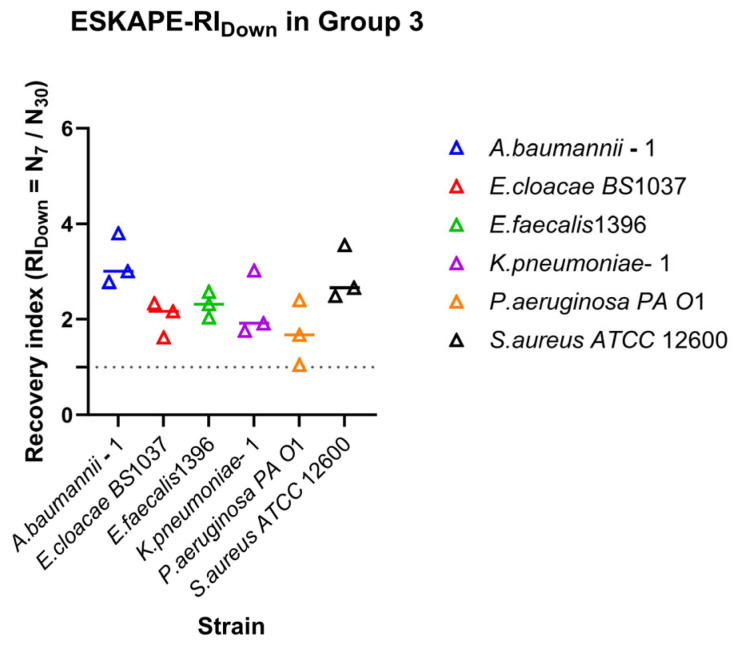
Recovery of surface-associated densities along the decreasing shear rates. Recovery indices (RI_Down_) were calculated from surface-associated densities measured along the 30–15–7 s^−1^ shear rate. Individual points represent biological replicates, while the horizontal line represents the median value. The dashed horizontal line indicates RI = 1, corresponding to no change in surface-associated bacterial densities between the final shear rate and the initial shear rate.

**Figure 7:**
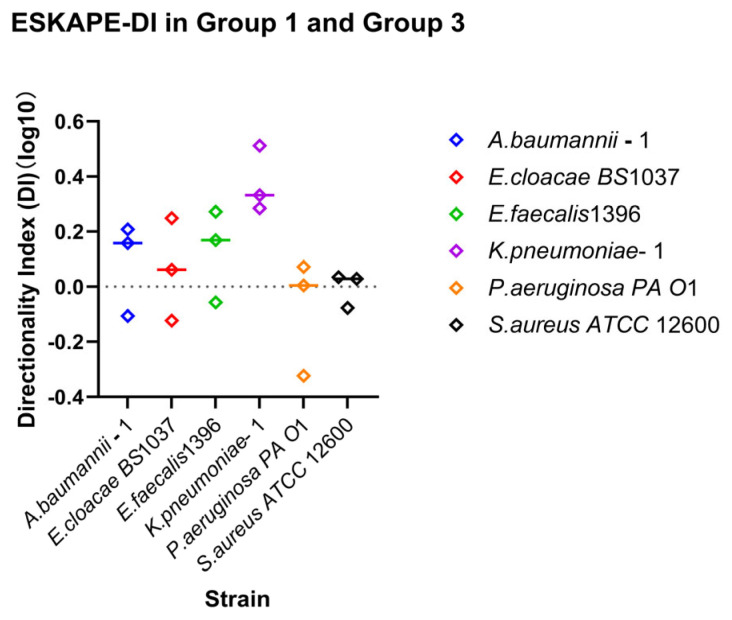
Relationship between shear direction and surface-associated bacterial density. Directionality indices (DI) were calculated for each strain as the ratio between recovery and retention indices (DI = RI_Down_/RI_30_). Individual points represent independent biological replicates; the horizontal line above the symbol represents the median value. Log_10_ DI values close to 0 indicate symmetric surface association density affinities for recovery and retention under increasing and decreasing shear rates. Values greater or less than 0 indicate direction-dependent surface association density affinities favouring recovery or retention, respectively.

Surface-associated bacterial densities under dynamic shear conditions were characterised by quantifying retention under increasing shear rates, recovery during decreasing shear rates, and direction-dependent surface-associated behaviour using retention indices (RI), recovery indices (RI_Down_), and the directionality index (DI) (Figure 5, Figure 6 and Figure 7). Retention of surface-associated bacterial density under moderate increasing shear rates (7–15–30 s^−1^) exhibited pronounced strain-specific differences (Figure 5a). For the moderately increasing shear rates, retention indices varied widely among strains. The *Staphylococcus aureus ATCC* 12600 showed high retention with RI values well above inflection (RI = 1), indicating the ability to continuously attach at higher shear rates. *Acinetobacter baumannii*–1, *Enterobacter cloacae BS* 1037, *Pseudomonas aeruginosa PA O*1, and *Enterococcus faecalis* 1396 displayed moderate retention. In contrast, *Klebsiella pneumoniae*–1 exhibited retention indices close to RI = 1, indicating near equal surface association and disassociation events under increasing shear rates.

When the shear rate range was extended to higher values (15–30–60 s^−1^) (Figure 5b), overall retention tended to decrease, although the relative ordering of strains was largely conserved. Several strains consistently maintained RI values above inflection, whereas others exhibited RI values approaching inflection (Figure 5b), highlighting that retention under moderate shear did not necessarily predict retention under higher shear. Recovery of surface-associated bacterial density during decreasing shear also varied among strains (Figure 6). *Acinetobacter baumanii*–1 and *Staphylococcus aureus ATCC* 12600 exhibited high recovery indices, indicating strong restoration of surface associations following shear reduction. *Enterococcus faecalis* 1396, *Enterobacter cloacae BS* 1037, and *Klebsiella pneumoniae*–1 showed moderate recovery behaviour. *Pseudomonas aeruginosa PA O*1 also exhibited moderate recovery; however, one replicate approached RI = 1, suggesting limited recovery after exposure to high shear rates.

Direction-dependent surface-associated behaviour was further examined by comparing retention and recovery responses (Figure 7). Log_10_ DI values revealed clear strain-specific patterns. *Klebsiella pneumoniae*–1 stands out due to a lack of enhanced surface associations with increasing shear rate, but recovering surface associations under reducing shear rate. *Acinetobacter baumannii*–1, *Enterobacter cloacae BS* 1037, and *Enterococcus faecalis* 1396 also exhibited predominantly positive, but less drastic, log_10_ DI values, indicating stronger recovery relative to retention. In contrast, *Pseudomonas aeruginosa PA O*1 and *Staphylococcus aureus ATCC* 12600 displayed log_10_ DI values close to zero, suggesting largely symmetric affinity for retention and recovery under opposing directional shear rate changes.

### 3.4. Bond Strength to Glass for ESKAPE Panel Bacteria Adapting to Various Shear Rates Modelled via the Spring Constant

**Figure 8:**
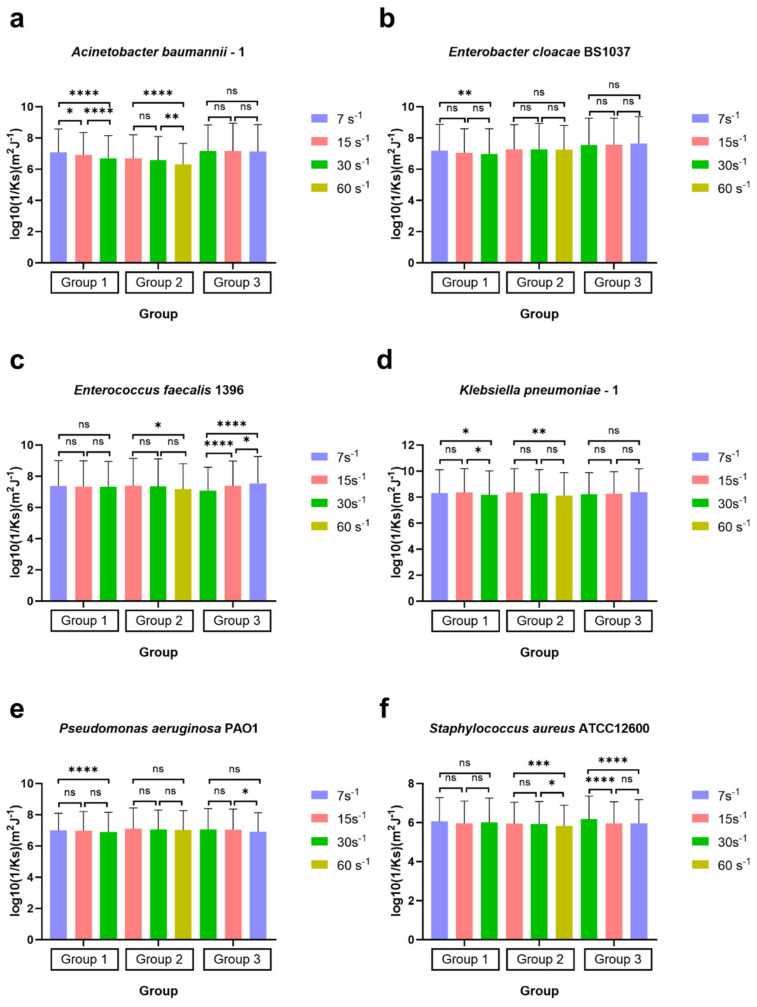
Surface-associated bond stiffness to glass represented by the reciprocal of the spring constant log_10_(1/Ks) of ESKAPE panel bacteria under the three sets of different shear rates. Six bacterial species *Acinetobacter baumannii*–1 (**a**), *Enterobacter cloacae* BS1037 (**b**), *Enterococcus faecalis* 1396 (**c**), *Klebsiella pneumoniae*–1 (**d**), *Pseudomonas aeruginosa* PA01 (**e**), and *Staphylococcus aureus* ATCC 12600 (**f**) on glass were exposed to three treatment groups according to the directional change in shear rate, namely group 1 (7 s^−1^→15 s^−1^→30 s^−1^), group 2 (15 s^−1^→30 s^−1^→60 s^−1^), and group 3 (30 s^−1^→15 s^−1^→7 s^−1^). The surface-associated bond stiffness is represented by the mean value of the log_10_ inverse spring constant, with error bars representing standard deviation (SD, specific N can be found in Table 3, range 421–2335). Individual bacteria that remained associated with the surface for 20 s were counted, representing 84% [range 73–92%] of all bacteria quantified as associated with the surface. Data were analysed using a one-way ANOVA with Tukey’s HSD post hoc test. Statistical significance is represented by * *p* < 0.05, ** *p* < 0.01, *** *p* < 0.001, **** *p* < 0.0001. Scientific relevance is represented by *p* < 0.001, and representative videos are uploaded to the Supplementary Materials.

The surface-associated bond strength to glass surfaces was modelled via time capture images from a phase contrast microscope (25 fps for 20 s) and centre of mass detection to determine displacement used in mean squared displacement (MSD) calculations. These calculations resulted in spring constants (Ks) between the bacterium and the glass surface, representing the stiffness of the general bacterial adhesion ligament. A larger Ks indicates greater stiffness and resistance to deformation, while a smaller Ks means increased softness [50]. Similar to surface-associated bacterial density, an ANOVA test with Tukey’s HSD post hoc test was employed to compare the log_10_ of the reciprocal of the spring constant log_10_ (1/Ks) for each bacterial strain, examining the differences in stiffness of bacterial surface-associated strength to a glass surface under varying shear rates (Figure 8).

In group 1 (7 s^−1^→15 s^−1^→30 s^−1^), the values of log_10_(1/Ks) for four bacterial strains, *Acinetobacter baumannii*–1 (Figure 8a), *Enterobacter cloacae BS* 1037 (Figure 8b), *Klebsiella pneumoniae*–1 (Figure 8d), and *Pseudomonas aeruginosa PA O*1 (Figure 8e), significantly decreased as the shear rate increased. This trend indicates a corresponding rise in the value of Ks, suggesting that the adhesion tethers of these strains became more resistant to compression and stretching, resulting in greater stiffness. *Klebsiella pneumoniae*–1 and *Enterobacter cloacae BS* 1037 had *p*-values > 0.001, which we will interpret as not scientifically relevant due to the high N. Notably, the analysis observed significant differences in log_10_(1/Ks) values among the strains at the initial shear rate of 7 s^−1^ compared to the final rate of 30 s^−1^.

*Acinetobacter baumannii*–1 (Figure 8a) also exhibited significant changes between the shear rates of 7 s^−1^ and 15 s^−1^ and between 15 s^−1^ and 30 s^−1^. In contrast, *Enterobacter cloacae BS* 1037 (Figure 8b) and *Pseudomonas aeruginosa PA O*1 (Figure 8e) displayed a slower increase in stiffness throughout the increase in shear rate without statistical differences between intermediate measurements. On the other hand, *Klebsiella pneumoniae–*1 (Figure 8d) revealed an additional significant difference between the shear rates of 15 s^−1^ and 30 s^−1^, suggesting the change in stiffness required an excess of 15 s^−1^ shear to activate. The spring constants of *Enterococcus faecalis* 1396 (Figure 8c) and *Staphylococcus aureus ATCC* 12600 (Figure 8f) did not change significantly in the first treatment group.

In group 2 (15 s^−1^→30 s^−1^→60 s^−1^), the log_10_(1/Ks) values for the four strains, *Acinetobacter baumannii–*1 (Figure 8a), *Enterococcus faecalis* 1396 (Figure 8c), *Klebsiella pneumoniae*–1 (Figure 8d), and *Staphylococcus aureus ATCC* 12600 (Figure 8f), decreased significantly as the shear rate increased. Among these strains, the log_10_(1/Ks) values had additional significance at between 30 s^−1^ and 60 s^−1^ for *Acinetobacter baumannii*–1 and *Staphylococcus aureus ATCC* 12600, while no significant differences were found at 15 s^−1^ and 30 s^−1^, indicating that the 60 s^−1^ shear rate had the most impact on these strains. In contrast, the spring constants for *Enterobacter cloacae BS* 1037 (Figure 8b) and *Pseudomonas aeruginosa PA O*1 (Figure 8e) showed no differences throughout the increase in shear rates. *Enterococcus faecalis* 1396 and *Klebsiella pneumoniae*–1 had *p*-values > 0.001, which we will interpret as not scientifically relevant due to the high N.

In group 3 (30 s^−1^→15 s^−1^→7 s^−1^) with decreasing shear rates, the log_10_(1/Ks) values for *Acinetobacter baumannii*–1 (Figure 8a), *Enterobacter cloacae BS* 1037 (Figure 8b), and *Klebsiella pneumoniae*–1 (Figure 8d) remained consistent with no significant changes from beginning to the end. *Pseudomonas aeruginosa PA O*1 (Figure 8e) also remained consistent as the shear rate gradually decreased with no significant changes from start to finish. Despite that, *Pseudomonas aeruginosa PA O*1 (Figure 8e) exhibited a significant increase (*p* < 0.05) in stiffness when the shear rate changed from 15 s^−1^ to 7 s^−1^; however, this could be an artefact of the high N and thus deemed not scientifically relevant. Only two strains, *Enterococcus faecalis* 1396 (Figure 8c) and *Staphylococcus aureus ATCC* 12600 (Figure 8f), displayed noteworthy variations in stiffness (*p* < 0.001) throughout the experiment. *Enterococcus faecalis* 1396 demonstrated a significant decrease in stiffness as the shear rate was adjusted. In contrast, *Staphylococcus aureus ATCC* 12600 showed a significant increase in stiffness.

### 3.5. Bond Strength to Glass for ESKAPE Panel Bacteria Adapting to Similar Shear Rates Modelled via the Spring Constant

**Figure 9:**
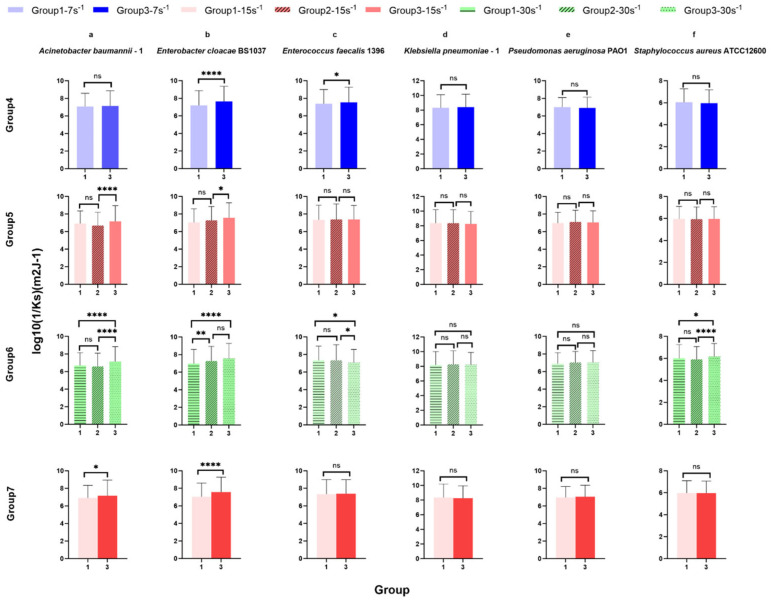
Surface-associated bond stiffness to glass represented by the reciprocal of the spring constant log_10_(1/Ks) of ESKAPE panel bacteria at the same shear rate for control analysis. Six bacterial species *Acinetobacter baumannii*–1 (**a**), *Enterobacter cloacae* BS1037 (**b**), *Enterococcus faecalis* 1396 (**c**), *Klebsiella pneumoniae*–1 (**d**), *Pseudomonas aeruginosa* PA01 (**e**), and *Staphylococcus aureus* ATCC 12600 (**f**) on glass were exposed to three treatment groups according to the directional change in shear rate, namely group 1 (7 s^−1^→15 s^−1^→30 s^−1^), group 2 (15 s^−1^→30 s^−1^→60 s^−1^), and group 3 (30 s^−1^→15 s^−1^→7 s^−1^). The data were further divided into three groups (rows, groups 4–7) according to the same shear rate, namely group 4 (7 s^−1^), group 5 (15 s^−1^), and group 6 (30 s^−1^). Group 7 showed the bacterial density of six bacterial strains at the same shear rate (15 s^−1^) and the same time (intermediate). The adhesion bond stiffness is represented by the mean value of the log_10_ inverse spring constant, with error bars representing standard deviation (SD, specific N can be found in Table 3, range 421–2335). Individual bacteria that remained associated with the surface for 20 s were counted, representing 84% [range 73–92%] of all bacteria quantified as associated with the surface. Data were analysed using a one-way ANOVA with Tukey’s HSD post hoc test. Statistical significance is represented by * *p* < 0.05, ** *p* < 0.01, **** *p* < 0.0001. Scientific relevance is represented by *p* < 0.001.

Using the same ANOVA and Tukey’s HSD post hoc tests in groups 1–3, thus controlling for multiple comparisons, four additional control assessments were compared to determine the influences of potential confounding variables. In group 4 (Figure 9) [7 s^−1^ shear rate between treatment group 1 (un-adapted: 7 s^−1^→15 s^−1^→30 s^−1^) and group 3 (reduced shear: 30 s^−1^→15 s^−1^→7 s^−1^)], both *Enterobacter cloacae BS* 1037 (Figure 9b group 4) and *Enterococcus faecalis* 1396 (Figure 9c group 4) exhibited a significant decrease in their spring constant. *Enterobacter cloacae BS* 1037 had the only significance deemed scientifically relevant.

Under 15 s^−1^ in group 5 [group 1, intermediate-up (7 s^−1^→15 s^−1^→30 s^−1^), group 2, initial (15 s^−1^→30 s^−1^→60 s^−1^), and group 3 intermediate-down (30 s^−1^→15 s^−1^→7 s^−1^)], the spring constant of *Acinetobacter baumannii*–1 (Figure 9a group 5) *and Enterobacter cloacae BS* 1037 (Figure 9b group 5) significantly decreased between treatment groups 2 and 3, with *A. baumannii* having the only scientifically relevant difference. The spring constants of more bacterial strains were significantly affected in group 6 [group 1, final (7 s^−1^→15 s^−1^→30 s^−1^), group 2, intermediate (15 s^−1^→30 s^−1^→60 s^−1^), and group 3, initial (30 s^−1^→15 s^−1^→7 s^−1^)] at a higher shear rate of 30 s^−1^. The spring constants of *Acinetobacter baumannii*–1 (Figure 9a group 6), *Enterobacter cloacae BS* 1037 (Figure 9b group 6), and *Staphylococcus aureus ATCC* 12600 (Figure 9f group 6) significantly decreased between treatment groups 1 and 3. Additionally, *A. baumannii*–1 and *S. aureus ATCC* 12600 significantly decreased in the spring constant between treatment groups 2 and 3, while *E. cloacae BS* 1037 significantly decreased in the spring constant between treatment groups 1 and 2. Conversely, the spring constant of *Enterococcus faecalis* 1396 (Figure 9c group 6) significantly increased between treatment groups 1 and 3 and between treatment groups 2 and 3. *Klebsiella pneumoniae*–1 (Figure 9d group 6) and *Pseudomonas aeruginosa PA O*1 (Figure 9e group 6) did not exhibit significant changes in stiffness. *E. faecalis* 1396 did not have scientifically relevant differences.

Group 7 holds residence time and shear rate constant [group 1, increasing (7 s^−1^→15 s^−1^→30 s^−1^) and group 3, decreasing (30 s^−1^→15 s^−1^→7 s^−1^)]. Within this group, *Acinetobacter baumannii*–1 (Figure 9a group 7) and *Enterobacter cloacae BS* 1037 (Figure 9b group 7), show a significant decrease in their spring constants when adapting from a higher shear rate compared to adapting from a lower shear rate. *E. cloacae* had the only scientifically relevant difference.

In general, both increasing time elapsed and decreasing shear rate promoted an increase in surface-associated bacterial density to glass. Time elapsed was more influential at lower shear rates than at shear rates above 15 s^−1^. Increasing the shear rate also increased the stiffness of the surface-associated bond, as measured by the spring constant (Ks), but was species-specific as to the intensity of shear rate required to enhance stiffening. Decreasing the shear rates generally did not influence the stiffness of the bond; however, two species displayed opposite stiffening effects under these conditions. In contrast to bacterial density, stiffening displayed more influence with the higher shear rate (30 s^−1^) than with the lower shear rates. *E. cloacae* were the only bacteria to show a significant difference in stiffness when time and shear were constant, confirming adaptation from residual energy from the previously seen shear rate.

### 3.6. Strain-Specific Surface-Associated Bond Strengths Under Directional Dynamic Shear

**Figure 10:**
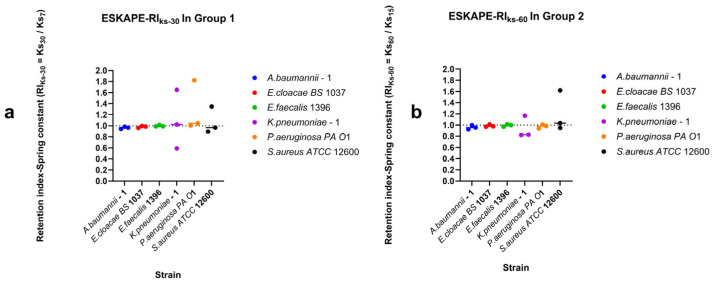
Retention of surface-associated spring constants along increasing shear rates. Retention indices were calculated from surface-associated bond strengths measured for Group 1 (7–15–30 s^−1^ shear rates (**a**)) and Group 2 (15–30–60 s^−1^ shear rates (**b**)). Individual points represent the geometric mean spring constant per biological replicate as a single data point estimate. The horizontal line per species represents the median value, and the dashed horizontal line indicates RI = 1, corresponding to no change in surface-associated spring constants between the final shear rate and the initial shear rate.

**Figure 11:**
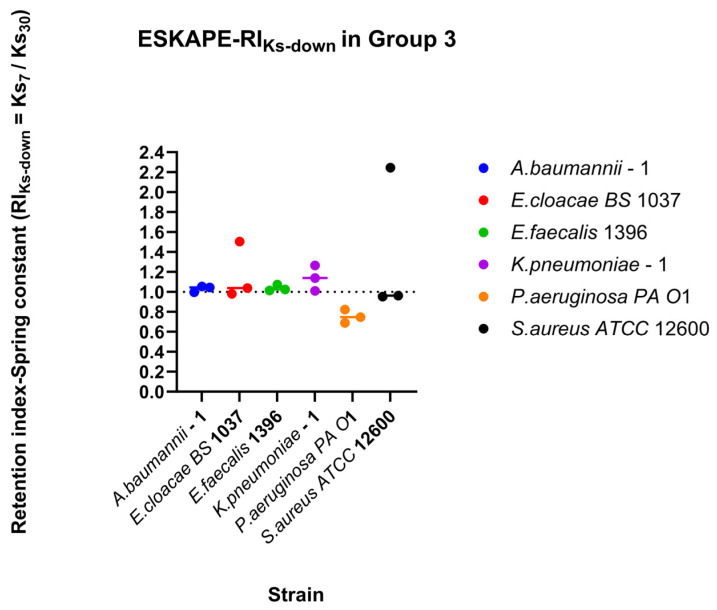
Recovery of surface-associated spring constants along the decreasing shear rates. Recovery indices (RI_Down_) were calculated from surface-associated strength measured for Group 3 (30–15–7 s^−1^ shear rates). Individual points represent the geometric mean spring constant per biological replicate as a single data point estimate. The horizontal line per species represents the median value, and the dashed horizontal line indicates RI = 1, corresponding to no change in surface-associated spring constants between the final shear rate and the initial shear rate.

**Figure 12:**
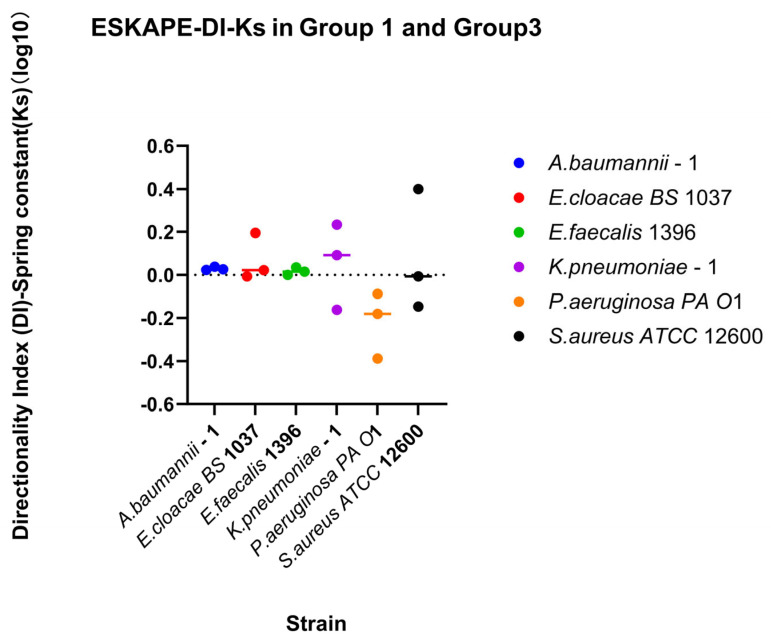
Relationship between changing shear rate direction and spring constant. Direction-dependent surface association strength was assessed using the directionality index. Directionality indices (DI) were calculated for each biological replicate for each shear rate condition as the ratio between recovery and retention indices (DI-Spring constant = RI_Down-Ks_/RI_30-Ks_). Individual points represent the log_10_ DI for each biological replicate and shear rate condition. The horizontal line above the symbol represents the median value. A reference line at log_10_ DI = 0 represents symmetric surface association strength outcomes under increasing and decreasing shear trajectories, whereas values greater or less than 0 indicate direction-dependent attachment behaviour favouring recovery or retention, respectively.

Surface-associated bond strengths under dynamic shear conditions were characterised by quantifying retention under increasing shear rates, recovery during decreasing shear rates, and direction-dependent surface-associated behaviour using retention indices (RI), recovery indices (RI_Down_), and the directionality index (DI) (Figure 10, Figure 11 and Figure 12). Retention Indices for all strains remained close to the inflection point (RI = 1) for all bacterial strains tested (Figure 10), except for *Klebsiella pneumoniae*–1, which, under higher shear rates, has a higher spring constant (stiffer bond) at the lower shear rate (15 s^−1^) than at the higher shear rate (60 s^−1^) (Figure 10b). The recovery index (RI_Down_) shows a similar trend, with most spring constants at the reflection point RI = 1. *Klebsiella pneumoniae*–1 again has a higher spring constant at higher shear rates, but *Pseudomonas aeruginosa PA O*1 has a higher spring constant at lower shear rates (Figure 11).

**Figure 13:**
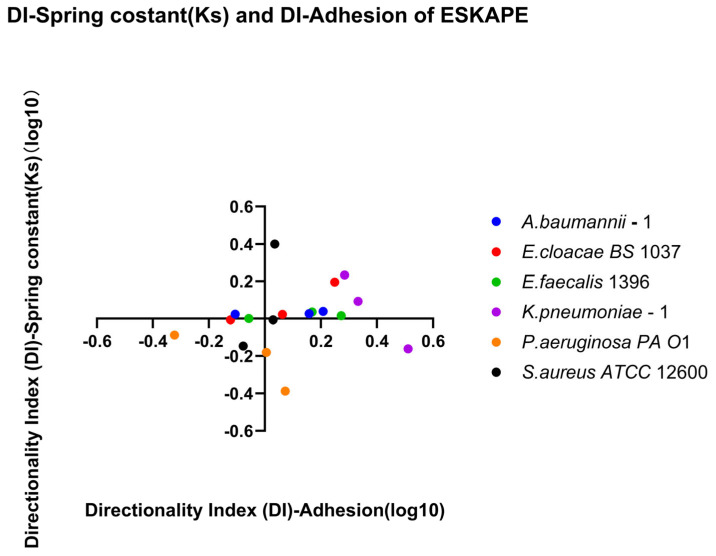
Quadrant analysis of affinity for retention versus recovery. Scatter plot showing the relationship between the directionality index derived from surface-associated density (DI-Adhesion) and the directionality index derived from spring constant (DI-Spring constant (Ks)) across six bacterial strains per biological replicate and each shear rate condition. Individual points represent the log_10_ DI for each biological replicate and shear rate condition. Quadrant I (top left, log_10_ DI-adhesion < 0, log_10_ DI-spring constant > 0), Quadrant II (top right, log_10_ DI-adhesion > 0, log_10_ DI-spring constant > 0), Quadrant III (bottom left, log_10_ DI-adhesion < 0, log_10_ DI-spring constant < 0), and Quadrant IV (bottom right, log_10_ DI-adhesion > 0, log_10_ DI-spring constant < 0) are defined to reflect affinities for retention versus recovery. Positive DI values indicate increased affinity for surface-associated bacterial densities or bond strengths, whereas negative DI values indicate reduced affinity for surface-associated bacterial densities or bond strengths.

Direction-dependent surface-associated bond strength behaviour was further examined by comparing retention and recovery responses (Figure 12). *Klebsiella pneumoniae*–1 has a small affinity for recovery, while *Pseudomonas aeruginosa PA O*1 has a small affinity for retention of its spring constants. Comparing direction indices between the two measured parameters, a majority of biological replicates fall, favouring recovery or retention for both parameters (Quadrants II and III, Figure 13). Looking at each bacterial strain independently, three strains have sizable differences in one parameter while barely changing in the other. The other three bacterial strains change for both parameters across bacterial replicates.

## 4. Discussion

This study aimed to investigate how varying low shear rates (by gradually increasing 7–15–30 s^−1^, 15–30–60 s^−1^, and decreasing 30–15–7 s^−1^) affect the adhesion of ESKAPE bacteria to a glass surface of a flow chamber. To avoid specific species influences on the conclusions, this study was performed with six diverse bacterial species, each represented by highly diverse known shape properties and known for resistance development.

### 4.1. Effect on Surface-Associated Bacterial Density

#### 4.1.1. Impact of Shear Rate Adaptation

For the same shear rate setting, the adhesion of the six bacterial species displayed varying trends. This variability is not only influenced by external environmental factors, such as the hydrodynamics, but is also closely linked to the inherent characteristics of the bacteria themselves.

Elapsed time and applied shear rate play significant roles in the bacterial adhesion process [51,52]. A previous study indicated that the main reason for the unchanged bacterial adhesion density might be a hydrodynamic blockage, as incoming cells are influenced by already adhered cells or the space on the surface [41]. It has been shown that the size of the blockage zone increases with higher shear rates and larger particle sizes. This observation may explain the significant increase in bacterial adhesion that occurs during the transition from high to low shear rates (Figure 3 group 3 a, b, f) as the blockage zone shrinks, allowing for more adhesion. Additionally, higher shear rates enhance interactions between planktonic cells and surfaces, but the increased shear forces can hinder adhesion or promote detachment [53,54]. As the shear rate increases, the wall shear rate also rises, leading to greater drag forces. Nejadnik et al. have shown that when shear rates reach sufficiently high levels, adhering bacteria can slide and roll over a surface, which may cause them to detach [55]. Disassociation events to the surface occurring with relatively equal new associations to the surface from the bacterial suspension could explain why generally the surface-associated bacterial density did not change with increasing shear rate (Figure 3 group 1. a, b, c, d; Figure 3 group 2. a, b, d, e, f).

#### 4.1.2. Bacterial Shape and Shear Rate Adaptation

In addition to variations in shear rates, different bacterial species exhibit distinct adhesion patterns on surfaces. For instance, the shape of bacterial cells affects their ability to attach, as they adhere to solid surfaces through van der Waals forces and electrostatic interactions [56,57,58]. Cocci bacteria contact the flat surface at a small point, whereas rod-shaped bacteria can touch the same surface along a linear set of points that extends the length of the cell [59,60]. *S. aureus*, which is spherical, has an average diameter of about 1 µm [59], while rod-shaped *P. aeruginosa* measures approximately 1 µm in diameter and 5 µm in length [60]. The volume of *P. aeruginosa* is about 7.5 times larger than the volume of *S. aureus* [59,60]. Similarly, the low shear rate increased the surface-associated bacterial density of *S. aureus ATCC* 12600 (Figure 3f group 1) cells on the surface. Although not tested, viability also can play a role in bacterial attachment strength to the surface [61,62].

The increase in shear rate will enhance the shear force on bacteria adhering to the glass surface. The strength of the parallel component of surface shear is proportional to the square of the radius of the particle [58,63], and it determines if a cell remains attached or is removed. A coccal bacterium, due to its spherical shape, presents the same surface area to any fluid flow, regardless of how the cell is positioned. In contrast, a rod-shaped cell can align itself lengthwise with the direction of the fluid flow, allowing it to interact differently with the surrounding environment. Theoretically, rod-shaped cells should be capable of enduring greater shear stress if they are arranged longitudinally in the orientation of fluid flow, compared to the same mass oriented differently. For example, when subjected to high shear force, *E. coli* elongates without a significant change in diameter and is more likely to grow in chains [64]. These reactions expand the surface area available for adhering while keeping the cross-sectional area that is susceptible to shear forces constant. As not all bacteria may be possible to align with fluid flow, *P. aeruginosa* generally experiences higher fluid dynamic drag due to its size and shape. However, the higher potential contacts with the surface due to size and geometry help explain why the surface-associated density of *P. aeruginosa PA O*1 (Figure 3e group 2 and group 3) and possibly *Klebsiella pneumoniae*–1 (Figure 3d) on glass surfaces is stable with the higher fluid shear rate.

Rod-shaped bacteria have additional strategies to enhance their adhesion ability. *B. subtilis* can shrink its cross-sectional area, decreasing to one-fourth of its original value, reducing total shear under high-shear conditions [65]. A similar phenomenon may be reflected in our experiments, as the surface-associated bacterial densities of *Enterobacter cloacae BS* 1037 (Figure 3b group 2 from 15 s^−1^ to 60 s^−1^) and *Pseudomonas aeruginosa PA O*1 (Figure 3e group 1) increased as the shear rate increased. Therefore, rod-shaped cells have a competitive advantage in environments with significant shear forces.

However, like any physiological trade-off, these points assume that all other factors are equal, which may not always be true. There are various other methods to enhance attachment, and cell shape may not always be the primary factor in this process, such as bacteria can respond to external mechanical stimuli through complex mechanisms to regulate their interactions with surfaces [54], or within a certain range, the “catch bond” mechanism expressed by *E. coli* enhances the bacteria’s ability to adhere [66]. The adhesion mechanism of *K. pneumoniae* might thus be more “static” (based on specific adhesin–surface binding, capsule interactions) and less reliant on shear-induced geometry changes or force-activated attachment [67,68]. And more recent work found that the higher shear forces help *Pseudomonas aeruginosa* counteract the type IV-pilus-retraction-driven tilting of the cell away from the surface, thus promoting a more stable adhered geometry [54]. In addition, *Staphylococcus aureus* has surface adhesins like Protein A (SpA) and von Willebrand factor-binding protein (vWbp) that bind to von Willebrand factor (vWF). Under shear stress, vWF stretches and reveals binding sites, reinforcing the SpA/vWbp–vWF interaction, which enhances bacterial adhesion to surfaces with increased shear or tensile force [69]. Each of these mechanisms triggers under different shear rate conditions. Therefore, in this experiment, the three different bacilli (*Enterobacter cloacae BS* 1037, *Klebsiella pneumoniae*–1, and *Pseudomonas aeruginosa PA O*1) and the two cocci (*Enterococcus faecalis* 1396 and *Staphylococcus aureus ATCC* 12600) exhibited different adhesion trends under increasing shear rate change trend.

From Figure 4 (group 4. a, b, c and group 6. f), the surface-associated bacterial density may also be related to the length of residence time at the same shear rate. The reason may be attributed to the continuous exposure to the bacterial suspension during the experiments.

The observation that the surface-associated bacterial density remains constant at the same shear rate over different periods may be due to bacteria occupying available surface locations during the initial attachment phase. As these locations become saturated, they create hydrodynamic blockage as incoming cells are repelled by already adhered cells or are unable to find available space on the surface not protected by hydrodynamic drag [41].

### 4.2. Surface-Associated Bacterial Density as a Direction-Dependent Process Under Fluctuating Shear

By integrating retention, recovery, and directionality analyses, this study demonstrates that surface-associated bacterial density under flow is highly strain-specific and strongly influenced by the direction of shear rate changes. Retention, which reflects the experimental condition under the higher end shear rate compared to the lower initial shear rate, revealed that strains differ markedly in their ability to withstand escalating hydrodynamic shear. Recovery reflects the experimental condition under which the lower end shear rate is compared to the higher initial shear rate and highlights distinct capacities to re-establish surface association following high-shear rate exposure.

Importantly, retention and recovery did not scale uniformly across shear ranges or strains, indicating that surface association stability cannot be inferred from a single shear rate condition. Instead, surface association outcomes depend on both the magnitude of shear rate and its history. This is likely due to differences in cell wall deformation and viscoelastic failure points of surface-associated “tethers” across the different bacterial strains [49,70]. The directionality index (DI) provided a compact framework to assess the relative affinity to recovery and retention responses under opposite changing shear rate directions.

Strains exhibiting positive log_10_DI values can be described as shear-adaptive, as they display enhanced recovery following shear rate reduction relative to their ability to retain during shear rate escalation. Conversely, strains with negative log_10_DI values exhibited shear-tolerant tendencies, characterised by stronger retention under increasing shear rates relative to their limited recovery once shear rate was reduced. Strains with log_10_DI values close to zero occupied an intermediate position, displaying largely symmetric recovery and retention. These phenotypes should be interpreted as positions along a continuum of early attachment strategies rather than as discrete categories, as they are measured in comparison with each other at the specific shear rates tested (30 s^−1^ and 7 s^−1^). Stronger shear rates may have more impact on the viscoelastic nature of surface association and change the observed relationships between retention and recovery.

Given that retention, recovery, and directionality indices were derived from a limited number of independent experimental replicates, these metrics are best interpreted as descriptive effect-size measures rather than as definitive quantitative properties. Nevertheless, the combined RI and DI framework highlights that surface association under dynamic flow conditions is governed not only by resistance to detachment but also by the capacity for reattachment following hydrodynamic challenge. Together, these findings underscore the importance of considering both shear rate magnitude and direction when assessing early surface colonisation under fluctuating flow environments.

### 4.3. Effect on the Spring Constant

This study reports the bacterial spring constants related to the mechanical features of bacterial cell surface association on glass surfaces for six different bacterial species, consisting of both Gram-positive and Gram-negative strains. Comparing different species with different cell wall structures raises the question of whether the outer membrane of a bacterium plays a specific role in the Brownian motion or confined Brownian motion of surface-associated bacteria.

To answer this question, this study performed experiments with four Gram-negative bacterial strains, *Klebsiella pneumoniae*–1, *Acinetobacter baumannii*–1, *Pseudomonas aeruginosa PA O*1, and *Enterobacter cloacae BS* 1037, and two Gram-positive bacterial strains, *Enterococcus faecalis* 1396 and *Staphylococcus aureus* ATCC 12600.

Upon exposure to gradually increasing shear rates (Groups 1 and 2), as shown in Figure 8, the spring constants of the six bacterial species showed a highly consistent pattern across species, notwithstanding some differences in their detailed responses. In four bacterial species (excluding *A. baumannii* and *E. faecalis*), only one of the two groups with increasing shear rates (Group 1 and Group 2) exhibited statistically significant results. Having only one group exhibit statistical significance is a reflection on the viscoelastic nature of the surface association. Once the viscoelastic failure point is reached (may require stronger shear rates than 60 s^−1^ for *E. faecalis*), the inability to recover creates significant changes to the strength of the surface association. Statistical significance with *p* < 0.05 and *p* ≥ 0.001 was not considered scientifically relevant due to the high N that may increase the chances of overinterpretation.

The viscoelastic nature is reflected in the measured spring constant because the fluid shear force acts on the surface-associated bacteria along the flow direction, and the combination of bacteria and the substrate surface is stretched, causing the elasticity of the “tether” to decrease and become harder, which is similar to the observed nanomechanical behaviour of *Lactobacillus* under shear [71]. Under decreasing shear rate, the spring constants of the four Gram-negative bacteria did not change significantly, but the spring constants of the two Gram-positive bacteria changed significantly (*p* < 0.001). The Ks of *Enterococcus faecalis* 1396 (Figure 8c) decreased with the decreased shear rate, but the spring constant of *Staphylococcus aureus ATCC 12600* (Figure 8f) increased with the decreased shear rate. A possible explanation could be a difference in the type of surface-associated tether. In streptococci, the polyelectrolyte network is formed by fibrillar surface appendages, and spring constants decrease with increasing fibrillar density on the bacterial surface [72,73]. In staphylococci, the higher the EPS, the higher the spring constants [74,75].

Figure 9 (group 4. b, c; group 5. a, b; group 6. c) shows that the stiffness of surface-associated bacteria decreases with time under three different shear rate conditions, as under sustained shear force, these structures begin to relax over time (reinforcing the viscoelastic rationale and depiction), which leads to a reduction in effective stiffness [26]. However, under the highest shear rate conditions, the stiffness of three strains of bacteria shows an increase with time (Figure 9 group 6. a, b, f). Under the influence of higher shear rates (30 s^−1^), the adhesion ability of these bacteria increases over time, the spring constant increases, and the connection structure becomes more rigid and stable. This may be because the surface proteins of these bacteria have the characteristics of catch bonds [76,77,78]. Within a certain range, the adhesion stability increases with the increase in shear force, which can better resist the effect of external shear force, thereby improving the survival ability of bacteria in a flow environment.

Under the shear rate of 15 s^−1^ and the same elapsed time, the spring constant of *Acinetobacter baumannii*–1 (Figure 9a group 7, *p* < 0.05, potentially not scientifically relevant) and *Enterobacter cloacae BS* 1037 (Figure 9b group 7, *p* < 0.0001) show a significant reduction between group 1 and group 3. This provides direct evidence of adaptation, as group 1 previously had a lower shear rate (7 s^−1^), while group 3 previously had a higher shear rate (30 s^−1^). Because the surface association can trigger bacterial structural adaptations that can redistribute stress, it can also reduce apparent stiffness. In some species, adhesins may uncoil, bending under load to increase contact area—this increases surface association [26].

### 4.4. Coupling Between Surface Association Behaviour and Mechanical Adaptation Under Dynamic Shear

An association between surface association and spring constant response to directional shear rate changes would suggest a partial coupling between phenotypic attachment behaviour and mechanical adaptation under dynamic shear. Bacteria may coordinate their surface attachment strategies with changes in mechanical properties to maintain adhesion.

Figure 13 demonstrates that bacterial surface association under dynamic shear conditions is shaped not only by the shear rates but also by the direction of shear rate changes. Shear rate direction-dependent modulation of bacterial surface association strength (spring constant), consistent with a mechanical-memory effect, is also demonstrated. Importantly, the dataset does not support a single monotonic relationship between stiffness and surface association bacterial density.

#### 4.4.1. Retention and Recovery Coupled with Surface-Associated Bacterial Density and Surface-Associated Strength (Spring Constant)

Retention and recovery are two independent indices that quantify the relationship between properties at the end shear rate condition versus the initial shear rate condition for increasing and decreasing shear rates, respectively. These indices were calculated and are depicted in Figure 5, Figure 6, Figure 10 and Figure 11; however, they do not consider possible affinity for retention or recovery. The directional index establishes any affinity for a single property in Figure 7 and Figure 12, yet this still lacks the capacity to observe connections between properties. Figure 13 plots the DI of surface-associated bacterial density against the DI of the surface-associated strength (spring constant) to observe potential connections between these adhesion properties.

In *Pseudomonas aeruginosa*, *Enterobacter cloacae*, and *Klebsiella pneumoniae*, changes in affinity to recovery were seen in both surface-associated bacterial density and surface-associated strength. Affinity for surface-associated bacterial density was primarily favoured for recovery, with *P. aeruginosa* having some biological replicates favouring retention. The surface-associated strength was species-specific, with *P. aeruginosa* favouring retention, *K. pneumoniae* mostly favouring recovery, and *E. cloacae* staying near equilibrium until a high affinity for recovery for surface-associated density made a smaller affinity for recovery for surface-associated strength visible. Caution should be applied when attempting to draw trend lines from these biological replicates; however, these three bacterial species would each have different regression line shapes, suggesting no general pattern exists.

The coupling of surface association bacterial density and surface association strength is consistent with a classical mechano-strengthening interpretation in which bacteria respond to shear-induced stress by increasing envelope rigidity, thereby improving resistance to deformation and detachment. Mechanistically, such stiffening could involve cytoskeletal reorganisation and coordinated peptidoglycan remodelling, both of which have been implicated in bacterial mechanical adaptation [79,80]. In addition, the magnitude of stiffening observed suggests that envelope stress-responsive regulatory pathways may become engaged in some conditions, potentially after viscoelastic failure of one or more “tethers”. One plausible candidate is the Rcs phosphorelay system, which responds to envelope perturbation and has been associated with mechanical and shear-related stress responses [81,82].

However, the dataset also indicates that surface-associated bacterial density and surface-associated strength are not coupled.

#### 4.4.2. Retention and Recovery Decoupled with Surface-Associated Bacterial Density and Surface-Associated Strength (Spring Constant)

In *Staphylococcus aureus*, *Acinetobacter baumanii*, and *Enterococcus faecalis*, affinity for either surface-associated bacterial density or surface-associated strength (spring constant) was independent of the other. *S. aureus* had various affinities for surface-associated strength, but remained near equal with affinity for surface-associated density. *E. faecalis* and *A. baumanii* demonstrated more affinity to recovery with surface-associated density but near equal affinity to surface-associated strength. It is interesting that these three bacteria have a coccoid description in their shape, with *A. baumanii* being unique as a coccoid-bacillus. In reflection of bacilli being capable of adjusting their cell structures to reduce hydrodynamic drag, this may allow them to alter affinity to both surface-associated bacterial density and surface-associated strength, whereas coccoid bacteria are not capable of adjusting to hydrodynamic drag and thus alter a single affinity for survival.

From a physical perspective, maintaining surface-associated density while weakening strength is consistent with a compliance-based mechanism. Reduced stiffness may increase bacterial cell wall surface conformity, enlarge the effective contact area, and enable more extensive engagement between bacterial adhesins and the substrate. Under flow, such increased contact can facilitate the transition from relatively limited contacts to more multivalent interactions, which are critical for resisting detachment [83]. This interpretation is further supported by evidence that, in ligand–receptor systems, deformability can enhance effective adhesion by improving geometric matching and bond recruitment, making softer interfaces functionally “stickier” under certain binding chemistries [84].

Theoretical and experimental studies indicate that bacterial cell walls can undergo plastic deformation during growth under mechanical load, providing a route toward long-lived changes in mechanical state consistent with mechanical memory [85]. This memory, demonstrated herein, is essential to consider when discussing bacterial adhesion, as it can significantly change the underlying properties when compared to bacteria previously not exposed to memory-inducing conditions.

Collectively, the data support a mechano-adaptive repertoire in which bacteria can enhance retention through either surface-associated stiffening or surface-associated bacterial density (strength in numbers). The preferred regime is species-specific and likely determined by the interaction between envelope mechanics, surface microtopography, and binding chemistry, providing a mechanistic basis for understanding why bacterial attachment outcomes under shear can vary strongly across environments.

## 5. Conclusions

Bacterial adhesion to contact materials can even serve as an initial step for further contamination in medical devices and infection in patients by secondary pathogens [86]. Our study demonstrates that directionally changing low hydrodynamic conditions have an impact on surface associations of ESKAPE panel bacteria. As shear rates change, the dynamics of bacterial surface association also fluctuate, affecting the bacterial density on surfaces. Higher shear rates tend to disrupt weak associations, leading to no increase in bacterial density, as new associations replace the disrupted associations. Lower shear rates facilitated increased associations. The density of bacteria associated with the surface stabilised under gradually increasing low shear rate conditions, indicating that the bacteria were also continuously adapting to the changing environment, whereas, under decreasing shear rates, more of the surface became available via fluid dynamics, and increased surface-associated densities were observed.

In addition, how firmly the bacteria were associated with the surface, as measured by the spring constant, was influenced by the change in shear rate for specific bacterial species. As the shear rate increases, the spring constant of bacteria also tends to rise, indicating increased association strength, as bacteria adjust to greater physical stress that can displace them from surfaces. Additionally, the spring constant exhibits three distinct trends as the shear rate decreases. For the four Gram-negative bacteria, the spring constant remains unchanged. For *Enterococcus faecalis* 1396, the spring constant decreases, while for *Staphylococcus aureus ATCC* 12600, the spring constant increases. The difference in cell walls between Gram-negative and Gram-positive bacteria is demonstrated here with adaptability to surface association strength under decreasing shear.

We demonstrate that retention and recovery are different methods of adaptation to environmental conditions utilised by different bacterial species. These adaptations may form the basis of upregulation and downregulation responses used for survival. Furthermore*,* affinity towards retention and recovery was species-specific but with underlying trends. Coccoid bacteria were able to change their affinity in either surface-associated bacterial density or surface-associated strength (spring constant) in response to directional changes in shear rates. However, bacilli were able to change their affinity for both properties during directional changes in shear rates.

These results demonstrate that bacterial adhesion under dynamic shear conditions is shaped not only by shear rate but also by directional changes in shear rates. We propose that this reflects dependent mechanical remodelling, consistent with a mechanical-memory effect. Importantly, the data show that mechanical changes alone are insufficient to predict surface-associated bacterial density outcomes and vice versa. This aligns with the “mechano-microbiology” framework, emphasising surface structure in mechanical interactions. Future studies should involve diverse bacterial strains and single-cell force spectroscopy to explore whether cellular polarity influences attachment geometry and mechanical responses. Understanding how bacteria detect and adapt to environmental changes is crucial for preventing pathogenic adhesion in medical devices. This mechanosensory capability enables bacteria to modify their adhesion properties, which is vital for developing strategies to reduce bacterial adhesion in medical and industrial contexts, ultimately aiding in the design of more resilient surfaces and improving infection control measures [87].

## Figures and Tables

**Table 1:** Bacterial strains, cell wall types, and shapes of six selected ESKAPE panel bacterial species. The six bacterial strains examined in this study belong to the group collectively referred to as ESKAPE pathogens [44], including *Enterococcus faecalis* 1396, *Staphylococcus aureus ATCC* 12600, *Klebsiella pneumoniae*−1, *Acinetobacter baumannii–*1*, Pseudomonas aeruginosa PA O*1, and *Enterobacter cloacae BS* 1037.

Bacterial Strain	Type of Gram	Shape
*Enterococcus faecalis* 1396	G+	coccus
*Staphylococcus aureus ATCC* 12600	G+	coccus
*Klebsiella pneumoniae*–1	G−	bacillus
*Acinetobacter baumannii*–1	G−	coccobacillus
*Pseudomonas aeruginosa PA O*1	G−	bacillus
*Enterobacter cloacae BS* 1037	G−	bacillus

Gram-positive is designated as G+, and Gram-negative is designated as G−.

**Table 3:** The number of bacteria analysed by videos of the spring constants.

Group Number		Biological Replicate Experiment Time		
	Bacterial Strain	First Time	Second Time	Third Time
Group 1 (7–15–30 s^−1^)	*Enterococcus faecalis* 1396	113,145,138	177,371,413	192,295,309
	*Staphylococcus aureus ATCC* 12600	203,431,617	409,675,779	228,377,503
	*Klebsiella pneumoniae*–1	302,284,277	192,235,184	225,249,139
	*Acinetobacter baumannii*–1	123,188,203	214,329,350	131,212,201
	*Pseudomonas aeruginosa PA O*1	144,203,275	159,250,277	400,413,508
	*Enterobacter cloacae BS* 1037	176,277,296	152,162,185	130,222,300
Group 2 (15–30–60 s^−1^)	*Enterococcus faecalis* 1396	210,334,386	232,359,348	318,358,383
	*Staphylococcus aureus ATCC* 12600	361,656,827	261,462,601	400,708,907
	*Klebsiella pneumoniae*–1	297,251,154	284,172,191	190,134,129
	*Acinetobacter baumannii*–1	94,128,134	158,158,143	169,231,277
	*Pseudomonas aeruginosa PA O*1	134,231,319	164,224,305	161,239,258
	*Enterobacter cloacae BS* 1037	151,208,176	160,221,248	217,328,351
Group 3 (30–15–7 s^−1^)	*Enterococcus faecalis* 1396	243,352,497	174,319,451	387,691,908
	*Staphylococcus aureus ATCC* 12600	134,301,456	329,662,903	285,398,570
	*Klebsiella pneumoniae*–1	250,509,531	145,239,167	314,243,341
	*Acinetobacter baumannii*–1	146,325,408	197,322,476	131,346,547
	*Pseudomonas aeruginosa PA O*1	126,213,271	276,369,445	162,266,151
	*Enterobacter cloacae BS* 1037	192,334,424	250,283,411	184,263,429

The three bacterial counts in each box correspond to the number of bacteria used in the shear rate analysis at each change in shear rate (initial, intermediate, and final) for each of three biological replicates per species and the Treatment Group (shear rate changes). For example, in Group 1 for *Enterococcus faecalis* 1396, the counts of 113, 145, and 138 correspond to the number of bacteria analysed for spring constants at 7, 15, and 30 s^−1^ for the first bacterial replicate. The second replicate for *Enterococcus faecalis* 1396 had the number of bacteria at 177, 371, and 413. The data for other groups can be obtained similarly. The number of bacteria analysed for spring constants was determined by the number of isolated bacteria in a confined window, where they were contained for a period of 20 s with no other bacteria entering the confined window. This amounted to an average of 84% of bacteria analysed for spring constants from the total counts collected, with a range of 73–92%.

## Data Availability

The datasets generated and analysed during the current study are stored on the secure institutional OneDrive of the University Medical Centre Groningen (UMCG). Due to institutional policies regarding data privacy and security, this repository is only accessible to authorised personnel within UMCG. However, the data that support the findings of this study are available from the corresponding author upon reasonable request.

## Supplementary Materials

The following supporting information can be downloaded at: https://www.mdpi.com/article/10.3390/microorganisms14030623/s1, Figure S1: Representative images for statistical significance of surface-associated bacterial densities; Figure S2: The Mean Squared Displacement of ESKAPE under different shear rates; and Video S1: Representative videos for scientifically significant surface-associated bond stiffness to glass.

## References

[1] Blackledge M.S., Worthington R.J., Melander C. (2013). Biologically inspired strategies for combating bacterial biofilms. Curr. Opin. Pharmacol..

[2] Rice L.B. (2008). Federal funding for the study of antimicrobial resistance in nosocomial pathogens: No ESKAPE. J. Infect. Dis..

[3] Pendleton J.N., Gorman S.P., Gilmore B.F. (2013). Clinical relevance of the ESKAPE pathogens. Expert. Rev. Anti Infect. Ther..

[4] De Oliveira D.M.P., Forde B.M., Kidd T.J., Harris P.N.A., Schembri M.A., Beatson S.A., Paterson D.L., Walker M.J. (2020). Antimicrobial Resistance in ESKAPE Pathogens. Clin. Microbiol. Rev..

[5] Chen S., Jiang Y., Wang W., Chen J., Zhu J. (2023). The effect and mechanism of iodophors on the adhesion and virulence of *Staphylococcus aureus* biofilms attached to artificial joint materials. J. Orthop. Surg. Res..

[6] Pietrocola G., Campoccia D., Motta C., Montanaro L., Arciola C.R., Speziale P. (2022). Colonization and Infection of Indwelling Medical Devices by *Staphylococcus aureus* with an Emphasis on Orthopedic Implants. Int. J. Mol. Sci..

[7] Bele Y.B., Thakare P.V., Ghanwate N.A. (2025). Adhesion study of *Pseudomonas aeruginosa* on different urinary catheter materials. Ethiop. J. Health Sci..

[8] Elzahaby D.A., Farrag H.A., Haikal R.R., Alkordi M.H., Abdeltawab N.F., Ramadan M.A. (2023). Inhibition of Adherence and Biofilm Formation of *Pseudomonas aeruginosa* by Immobilized ZnO Nanoparticles on Silicone Urinary Catheter Grafted by Gamma Irradiation. Microorganisms.

[9] Liedberg H., Lundeberg T. (1989). Silver coating of urinary catheters prevents adherence and growth of *Pseudomonas aeruginosa*. Urol. Res..

[10] Bixler G.D., Bhushan B. (2012). Biofouling: Lessons from nature. Philos. Trans. A Math. Phys. Eng. Sci..

[11] Ron E.Z., Dworkin M., Falkow S., Rosenberg E., Schleifer K.H., Stackebrandt E. (2006). Bacterial Stress Response. The Prokaryotes.

[12] Audia J.P., Webb C.C., Foster J.W. (2001). Breaking through the acid barrier: An orchestrated response to proton stress by enteric bacteria. Int. J. Med. Microbiol..

[13] Cavicchioli R., Thomas T., Curmi P.M. (2000). Cold stress response in Archaea. Extremophiles.

[14] Foster J.W., Spector M.P. (1995). How *Salmonella* survive against the odds. Annu. Rev. Microbiol..

[15] Hecker M., Völker U. (2001). General stress response of *Bacillus subtilis* and other bacteria. Adv. Microb. Physiol..

[16] Hengge-Aronis R. (2002). Recent insights into the general stress response regulatory network in *Escherichia coli*. J. Mol. Microbiol. Biotechnol..

[17] Poolman B., Blount P., Folgering J.H., Friesen R.H., Moe P.C., van der Heide T. (2002). How do membrane proteins sense water stress?. Mol. Microbiol..

[18] Stock A.M., Robinson V.L., Goudreau P.N. (2000). Two-component signal transduction. Annu. Rev. Biochem..

[19] Blount P. (2003). Molecular mechanisms of mechanosensation: Big lessons from small cells. Neuron.

[20] Demain A.L., Fang A. (2001). Secondary metabolism in simulated microgravity. Chem. Rec..

[21] Carniello V., Peterson B.W., van der Mei H.C., Busscher H.J. (2020). Role of adhesion forces in mechanosensitive channel gating in *Staphylococcus aureus* adhering to surfaces. NPJ Biofilms Microbiomes.

[22] Ingber D.E. (2003). Mechanosensation through integrins: Cells act locally but think globally. Proc. Natl. Acad. Sci. USA.

[23] Nickerson C.A., Ott C.M., Wilson J.W., Ramamurthy R., LeBlanc C.L., Höner zu Bentrup K., Hammond T., Pierson D.L. (2003). Low-shear modeled microgravity: A global environmental regulatory signal affecting bacterial gene expression, physiology, and pathogenesis. J. Microbiol. Methods.

[24] Rusconi R., Garren M., Stocker R. (2014). Microfluidics expanding the frontiers of microbial ecology. Annu. Rev. Biophys..

[25] Carniello V., Peterson B.W., van der Mei H.C., Busscher H.J. (2018). Physico-chemistry from initial bacterial adhesion to surface-programmed biofilm growth. Adv. Colloid. Interface Sci..

[26] Dufrêne Y.F., Persat A. (2020). Mechanomicrobiology: How bacteria sense and respond to forces. Nat. Rev. Microbiol..

[27] Laventie B.J., Jenal U. (2020). Surface Sensing and Adaptation in Bacteria. Annu. Rev. Microbiol..

[28] Persat A., Nadell C.D., Kim M.K., Ingremeau F., Siryaporn A., Drescher K., Wingreen N.S., Bassler B.L., Gitai Z., Stone H.A. (2015). The mechanical world of bacteria. Cell.

[29] Webster S.S., Wong G.C.L., O’Toole G.A. (2022). The Power of Touch: Type 4 Pili, the von Willebrand A Domain, and Surface Sensing by *Pseudomonas aeruginosa*. J. Bacteriol..

[30] Hansen C.E., Qiu Y., McCarty O.J.T., Lam W.A. (2018). Platelet Mechanotransduction. Annu. Rev. Biomed. Eng..

[31] Vollrath M.A., Kwan K.Y., Corey D.P. (2007). The micromachinery of mechanotransduction in hair cells. Annu. Rev. Neurosci..

[32] Hamill O.P., Martinac B. (2001). Molecular basis of mechanotransduction in living cells. Physiol. Rev..

[33] Johanson K., Allen P.L., Lewis F., Cubano L.A., Hyman L.E., Hammond T.G. (2002). *Saccharomyces cerevisiae* gene expression changes during rotating wall vessel suspension culture. J. Appl. Physiol..

[34] Klaus D.M., Bitton G. (2002). Space microbiology: Microgravity and microorganisms. Encyclopedia of Environmental Microbiology.

[35] Ingber D. (1999). How cells (might) sense microgravity. FASEB J..

[36] Berke A.P., Turner L., Berg H.C., Lauga E. (2008). Hydrodynamic attraction of swimming microorganisms by surfaces. Phys. Rev. Lett..

[37] Aprikian P., Tchesnokova V., Kidd B., Yakovenko O., Yarov-Yarovoy V., Trinchina E., Vogel V., Thomas W., Sokurenko E. (2007). Interdomain interaction in the FimH adhesin of *Escherichia coli* regulates the affinity to mannose. J. Biol. Chem..

[38] Dickinson R.B., Nagel J.A., McDevitt D., Foster T.J., Proctor R.A., Cooper S.L. (1995). Quantitative comparison of clumping factor- and coagulase-mediated *Staphylococcus aureus* adhesion to surface-bound fibrinogen under flow. Infect. Immun..

[39] Padron G.C., Shuppara A.M., Palalay J.S., Sharma A., Sanfilippo J.E. (2023). Bacteria in Fluid Flow. J. Bacteriol..

[40] Palalay J.S., Simsek A.N., Reed J.L., Koch M.D., Sabass B., Sanfilippo J.E. (2023). Shear force enhances adhesion of *Pseudomonas aeruginosa* by counteracting pilus-driven surface departure. Proc. Natl. Acad. Sci. USA.

[41] Busscher H.J., van der Mei H.C. (2006). Microbial adhesion in flow displacement systems. Clin. Microbiol. Rev..

[42] Palalay J.S., Sanfilippo J.E. (2025). Flow-induced bending of flagella controls bacterial surface behavior. Preprint. bioRxiv.

[43] Sjollema J., van der Mei H.C., Hall C.L., Peterson B.W., de Vries J., Song L., de Jong E.D., Busscher H.K., Swartjes J.J.T.M. (2017). Detachment and successive re-attachment of multiple, reversibly-binding tethers result in irreversible bacterial adhesion to surfaces. Sci. Rep..

[44] Mulani M.S., Kamble E.E., Kumkar S.N., Tawre M.S., Pardesi K.R. (2019). Emerging Strategies to Combat ESKAPE Pathogens in the Era of Antimicrobial Resistance: A Review. Front. Microbiol..

[45] Sjollema J., Busscher H.J., Weerkamp A.H. (1989). Real-time enumeration of adhering microorganisms in a parallel plate flow cell using automated image analysis. J. Microbiol. Meth.

[46] Osilla E.V., Marsidi J.L., Shumway K.R., Sharma S. (2023). Physiology, Temperature Regulation. StatPearls.

[47] Ansarizadeh M., Nguyen H.T., Lazovic B., Kettunen J., De Silva L., Sivakumar R., Junttila O., Rissanen S.-L., Hicks R., Singh P. (2025). Microfluidic vessel-on-chip platform for investigation of cellular defects in venous malformations and responses to various shear stress and flow conditions. Lab. Chip.

[48] Godain A., Vogel T.M., Fongarland P., Haddour N. (2023). Influence of Hydrodynamic Forces on Electroactive Bacterial Adhesion in Microbial Fuel Cell Anodes. Bioengineering.

[49] Ohmura T., Skinner D.J., Neuhaus K., Choi G.P.T., Dunkel J., Drescher K. (2024). In Vivo Microrheology Reveals Local Elastic and Plastic Responses Inside 3D Bacterial Biofilms. Adv. Mater..

[50] Song L., Sjollema J., Sharma P.K., Kaper H.J., van der Mei H.C., Busscher H.J. (2014). Nanoscopic vibrations of bacteria with different cell-wall properties adhering to surfaces under flow and static conditions. ACS Nano.

[51] Marshall B.T., Sarangapani K.K., Lou J., McEver R.P., Zhu C. (2005). Force history dependence of receptor-ligand dissociation. Biophys. J..

[52] Xu L.C., Vadillo-Rodriguez V., Logan B.E. (2005). Residence time, loading force, pH, and ionic strength affect adhesion forces between colloids and biopolymer-coated surfaces. Langmuir.

[53] Dickinson R.B., Cooper S.L. (1995). Analysis of shear-dependent bacterial adhesion kinetics to biomaterial surfaces. AICHE J..

[54] Lecuyer S., Rusconi R., Shen Y., Forsyth A., Vlamakis H., Kolter R., Stone H.A. (2011). Shear stress increases the residence time of adhesion of *Pseudomonas aeruginosa*. Biophys. J..

[55] Nejadnik M.R., van der Mei H.C., Busscher H.J., Norde W. (2008). Determination of the shear force at the balance between bacterial attachment and detachment in weak-adherence systems, using a flow displacement chamber. Appl. Environ. Microbiol..

[56] Rijnaarts H.H.M., Norde W., Bouwer E.J., Lyklema J., Zehnder A.J. (1995). Reversibility and mechanism of bacterial adhesion. Colloids Surf. B Biointerfaces.

[57] van Loosdrecht M.C., Lyklema J., Norde W., Zehnder A.J. (1990). Influence of interfaces on microbial activity. Microbiol. Rev..

[58] van Loosdrecht M.C., Lyklema J., Norde W., Zehnder A.J. (1989). Bacterial adhesion: A physicochemical approach. Microb. Ecol..

[59] Monteiro J.M., Fernandes P.B., Vaz F., Pereira A.R., Tavares A.C., Ferreira M.T., Rereira P.M., Veiga H., Kuru E., VanNieuwenhze M.S. (2015). Cell shape dynamics during the staphylococcal cell cycle. Nat. Commun..

[60] Diggle S.P., Whiteley M. (2019). Microbe Profile: *Pseudomonas aeruginosa*: Opportunistic pathogen and lab rat. Microbiology.

[61] Senevirathne S.W.M.A.I., Toh Y.-C., Yarlagadda P.K.D.V. (2022). Fluid Flow Induces Differential Detachment of Live and Dead Bacterial Cells from Nanostructured Surfaces. ACS Omega.

[62] Senevirathne S.W.M.A.I., Mathew A., Toh Y.C., Yarlagadda P.K.D.V. (2022). Bactericidal Efficacy of Nanostructured Surfaces Increases under Flow Conditions. ACS Omega.

[63] Powell M.S., Slater N.K. (1982). Removal rates of bacterial cells from glass surfaces by fluid shear. Biotechnol. Bioeng..

[64] Edwards N., Beeton S., Bull A.T., Merchunk J.C. (1989). A novel device for the assessment of shear effects on suspended microbial cultures. Appl. Microbiol. Biotechnol..

[65] Sahoo S., Verma R.K., Suresh A.K., Rao K.K., Bellare J., Suraishkumar G.K. (2003). Macro-level and genetic-level responses of *Bacillus subtilis* to shear stress. Biotechnol. Prog..

[66] Thomas W.E., Trintchina E., Forero M., Vogel V., Sokurenko E.V. (2002). Bacterial adhesion to target cells enhanced by shear force. Cell.

[67] Beckman R.L., Cella E., Azarian T., Rendueles O., Fleeman R.M. (2024). Diverse polysaccharide production and biofilm formation abilities of clinical *Klebsiella pneumoniae*. NPJ Biofilms Microbiomes.

[68] Schroll C., Barken K.B., Krogfelt K.A., Struve C. (2010). Role of type 1 and type 3 fimbriae in *Klebsiella pneumoniae* biofilm formation. BMC Microbiol..

[69] Viela F., Speziale P., Pietrocola G., Dufrêne Y.F. (2019). Bacterial pathogens under high-tension: *Staphylococcus aureus* adhesion to von Willebrand factor is activated by force. Microb. Cell.

[70] Carniello V., Peterson B.W., Sjollema J., Busscher H.J., van der Mei H.C. (2018). Surface enhanced fluorescence and nanoscopic cell wall deformation in adhering *Staphylococcus aureus* upon exposure to cell wall active and non-active antibiotics. Nanoscale.

[71] Tripathi P., Beaussart A., Alsteens D., Dupres V., Claes I., von Ossowski I., de Vos W.M., Palva A., Lebeer S., Vanderleyden J. (2013). Adhesion and nanomechanics of pili from the probiotic *Lactobacillus rhamnosus* GG. ACS Nano.

[72] Boulbitch A., Quinn B., Pink D. (2000). Elasticity of the rod-shaped gram-negative eubacteria. Phys. Rev. Lett..

[73] Sullan R.M.A., Beaussart A., Prachi T., Sylvie D., El-Kirat-Chatel S., Li J.K., Schneider Y.-J., Vanderleyden J., Lebeer S., Dufrêne Y.F. (2015). Single-cell force spectroscopy of bacterial pili. ACS Nano.

[74] Formosa-Dague C., Feuillie C., Beaussart A., Derclaye S., Kucharíková S., Lasa I., Van Dijck P., Dufrêne Y.F. (2016). Sticky matrix: Adhesion mechanisms in staphylococcal biofilms. Microb. Biotechnol..

[75] Otto M. (2008). Staphylococcal biofilms. Curr. Top. Microbiol. Immunol..

[76] Mathelié-Guinlet M., Viela F., Pietrocola G., Speziale P., Alsteens D., Dufrêne Y.F. (2020). Force-clamp spectroscopy identifies a catch bond mechanism in a Gram-positive pathogen. Nat. Commun..

[77] Viljoen A., Viela F., Mathelié-Guinlet M., Missiakas D., Pietrocola G., Speziale P., Dufrêne Y.F. (2021). *Staphylococcus aureus* vWF-binding protein triggers a strong interaction between clumping factor A and host vWF. Commun. Biol..

[78] Paiva T.O., Geoghegan J.A., Dufrêne Y.F. (2023). High-force catch bonds between the *Staphylococcus aureus* surface protein SdrE and complement regulator factor H drive immune evasion. Commun. Biol..

[79] Sun S.X., Jiang H. (2011). Physics of bacterial morphogenesis. Microbiol. Mol. Biol. Rev..

[80] White C.L., Gober J.W. (2012). MreB: Pilot or passenger of cell wall synthesis?. Trends Microbiol..

[81] Zietek M., Miguel A., Shi H., Khusainov I., Asmar A.T., Ram S., Wartel M., Sueki A., Schorb M., Goulian M. (2025). Bacterial cell widening alters periplasmic size and activates envelope stress responses. EMBO J..

[82] Miguel A., Zietek M., Shi H., Sueki A., Corona F., Maier L., Verheul J., den Blaauwen T., Van Valen D., Typas A. (2025). Modulation of bacterial cell size and growth rate via activation of a cell envelope stress response. mBio.

[83] Fang B., Gon S., Park M.H., Kumar K.N., Rotello V.M., Nüsslein K., Santore M.M. (2012). Using flow to switch the valency of bacterial capture on engineered surfaces containing immobilized nanoparticles. Langmuir.

[84] Liyanage S.H., Yan M. (2026). Bacterial adhesion on glyco-hydrogels: Impact of glycan and hydrogel stiffness. Colloids Surf. B Biointerfaces.

[85] Amir A., Babaeipour F., McIntosh D.B., Nelson D.R., Jun S. (2014). Bending forces plastically deform growing bacterial cell walls. Proc. Natl. Acad. Sci. USA.

[86] Bouhrour N., Nibbering P.H., Bendali F. (2024). Medical Device-Associated Biofilm Infections and Multidrug-Resistant Pathogens. Pathogens.

[87] Simões M., Simões L.C., Vieira M.J. (2010). A review of current and emergent biofilm control strategies. LWT-Food Sci. Technol..

