# Supplementary material for: Different Cell Wall Compositions of ESKAPE Isolates on Glass Surfaces Impact Adhesion Adaptability to Dynamic Shear Stress
Source: Microorganisms. 2026 Mar 10;14(3):623. doi: 10.3390/microorganisms14030623 (PMC13028879; doi:10.3390/microorganisms14030623)
Supplement: Supplementary file 1 [file microorganisms-14-00623-s001.zip › microorganisms-4148060-Supplemental Materials 03.02_BP.pdf]

Article

Different Cell Wall Compositions of ESKAPE Isolates on Glass Surfaces Impact Adhesion Adaptability to Dynamic Shear Stress

Zhuoyi Cui, Anje M. Slomp, Alesia V. Quiroga, Jelly Atema-Smit, Hans J. Kaper and Brandon W. Peterson *

Department of Biomaterials and Biomedical Technology, University Medical Centre Groningen, University of Groningen, 9713 AV Groningen, The Netherlands
* Correspondence: b.w.peterson@umcg.nl; Tel.: +31-50-361-61-10

Supplementary Materials

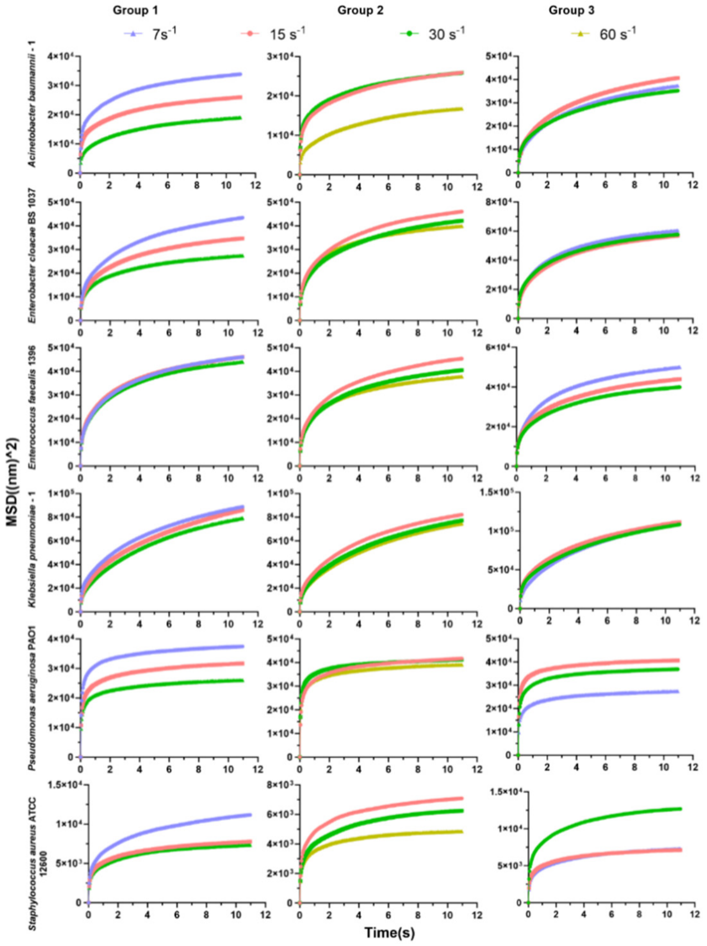

Figure S1. The Mean Squared Displacement of ESKAPE panel bacteria under different shear rates. The data of Mean Square Displacements were divided into three groups according to the change trend of shear rate, namely group 1 (7 s-1→15 s-1→30 s-1）, group 2 (15 s-1→30 s-1→60 s-1) and group 3 (30 s-1→15 s-1→7 s-1). The comparison results mainly compared the changes in the initial and final points of bacterial movement before and after the shear rate changed, which meant the starting and final points of the residence time for Mean Square Displacement.

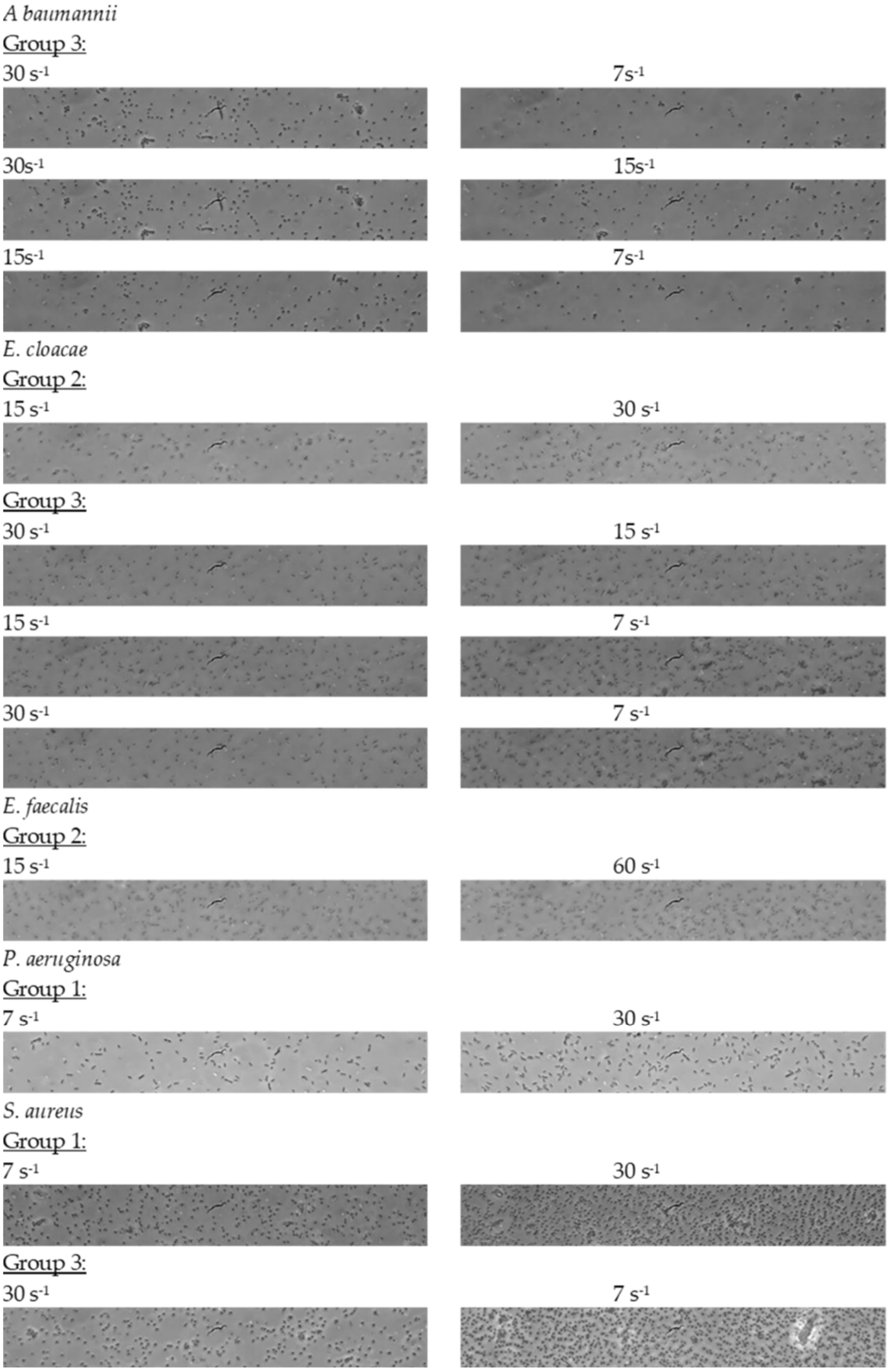

Figure S2. Representative images from scientifically relevant and statistically significant results in surface associated densities (adjusted +33% Brightness).

Representative videos from these scientifically relevant and statistically significant results in surface associated bond stiffness are uploaded separately in the supplemental materials.

A baumannii

Group 1:

7 s^{-1}	30 s^{-1}
15 s^{-1}	30 s^{-1}

E. faecalis

Group 3:

30 s^{-1}	15 s^{-1}
30 s^{-1}	7 s^{-1}

P. aeruginosa

Group 1:

7 s^{-1}	30 s^{-1}

S. aureus

Group 2:

15 s^{-1}	60 s^{-1}

Group 3:

30 s^{-1}	7 s^{-1}
30 s^{-1}	15 s^{-1}
